# Histones and nucleoid-associated proteins in *Methanosarcina acetivorans* mediate recovery from stress and stasis

**DOI:** 10.1128/spectrum.02785-26

**Published:** 2026-10-14

**Authors:** Alienor S. Baskevitch, Dipti D. Nayak

**Affiliations:** 1 Department of Plant and Microbial Biology, University of California 118549, Berkeley, California, USA; 2 Department of Molecular and Cell Biology, University of California 196203, Berkeley, California, USA; Connecticut Agricultural Experiment Station, New Haven, Connecticut, USA

**Keywords:** archaea, methanogen, *Methanosarcina*, histones, nucleoid-associated proteins

## Abstract

Many archaea encode histones in tandem with **n**ucleoid-**a**ssociated **p**roteins (NAPs), but the functional overlap between these two classes of non-specific **D**NA-**b**inding **p**roteins (DBPs) is not well studied. Most *in vivo* studies of DBPs have been conducted with archaea that only encode histones or NAPs. Here, we characterize the endogenous function of the two families of DBPs in a methanogen, *Methanosarcina acetivorans*, that encodes a histone and two copies of an archaea-specific NAP called **m**ethanogen **c**hromatin Protein 1 (MC1). In *M. acetivorans,* we found that each DBP is individually dispensable, but at least one copy of *mc1* appears to be essential. By and large, the single- and double-DBP knockouts phenocopy the wild-type strain under optimal laboratory growth conditions. Remarkably, mutants lacking the histone, one copy of MC1, or both recover faster than wild-type after being subjected to UV shock or long periods of growth stasis. Overexpression of the DBPs leads to a delay in growth recovery after extended periods in stationary phase, which is abrogated by introducing point mutations in DNA-binding residues. Together, our data suggest that histones and MC1 proteins have partially overlapping roles in *M. acetivorans*, one of which might be genome maintenance when growth has stalled or stopped.

**IMPORTANCE:**

Though it is known that many archaea encode histones in tandem with archaea-specific **n**ucleoid-**a**ssociated **p**roteins (NAPs), the interplay between these two classes of **D**NA-**b**inding **p**roteins (DBPs) *in vivo* is not known. Most studies on archaeal DBPs have focused either on archaea known to form histone-based chromatin or on strains that lack histones and compact their genomes exclusively with archaea-specific NAPs. Our study, therefore, fills an important gap in the literature by characterizing DBPs in a model methanogen, *Methanosarcina acetivorans*, which encodes both histones and archaea-specific NAPs. While no DBP is essential, at least one copy of MC1 appears to be needed, likely for genome compaction. In addition, mutants lacking DBPs had a growth advantage after being subjected to stressors or long periods of growth stasis, indicating that DBPs likely function in genome maintenance when growth has stalled or stopped.

## INTRODUCTION

Histones and nucleoid-associated proteins (NAPs) are two distinct classes of small, basic proteins that, by and large, non-specifically bind DNA (1, 2). Even though the canonical function of these proteins is to package and condense the genome, they also play other critical roles in the cell. In bacteria, NAPs induce topological changes in DNA—such as bending, bridging, and wrapping—that, besides chromosome compaction, are required for DNA protection, chromosome segregation, stress response, and gene regulation (2–4). In eukaryotes, histones are the primary constituents of chromatin and also play a central role in epigenetic regulation through post-translational modification of residues in their N-terminal tails (1, 5). In addition, the H3-H4 histone tetramer from eukaryotes has enzymatic activity and can function as a cupric reductase to produce bioavailable Cu^+1^ ions (6). Whether histones and NAPs also play such multifaceted roles in archaea remains unclear.

Unlike bacteria or eukaryotes, archaea lack a unified strategy for genome compaction. Some archaea encode histones, others encode NAPs, and many encode both classes of proteins (7–10). Archaeal histones are typically smaller than eukaryotic histones–most of them contain only the histone fold implicated in DNA binding and lack the N-terminal tails required for epigenetic regulation–but they share a common ancestor (9, 10). *In vivo* and *in vitro* studies show that histone homodimers from hyperthermophilic archaea such as *Thermococcus kodakerensis* and *Methanothermus fervidus* form nucleosome-like structures that are, indeed, involved in genome compaction (11–15). Even though histones are widespread within the archaea, they are not universally present, nor are they essential in mesophiles such as *Halobacterium salinarum* or *Methanosarcina mazei* Gö1 (16–18). These archaea also encode NAPs, which likely make histones dispensable. In some archaea, such as *Thermoplasma volcanicum*, histones have been replaced with a bacterial NAP, HU, which was likely acquired by horizontal gene transfer (8). In other lineages, new NAPs have been identified. One notable example is Alba (**a**cetylation **l**owers **b**inding **a**ffinity), a NAP that was initially discovered in the archaeon *Saccharolobus solfataricus* but is now found in all domains of life (19). In *S. solfataricus,* Alba associates with DNA to form filaments, which can then give rise to variable higher-order structures depending on the form(s) of Alba present and their expression level (20, 21). Alba is the most widespread NAP in archaea, and it often co-occurs with histones in thermophilic archaea within the *Thermococcales* and the *Archaeoglobales* (8). Besides Alba, **m**ethanogen **c**hromatin **p**rotein 1 (MC1) is another prominent archaeal NAP that frequently co-occurs with histones (8). By and large, MC1 tends to be encoded in archaeal lineages that lack Alba, and vice versa (8). Biochemical and structural studies with MC1 have shed light on its putative function *in vivo*. Conserved basic residues are critical for MC1-DNA interactions and lead to conformational changes in the DNA backbone (22–25). Upon binding DNA, MC1 induces bends of either 55° or 70°, and this bending action might have a variety of consequences (25). Induction of DNA kinks by MC1 is postulated to play a role in DNA protection and repair. This hypothesis is favored by studies in which MC1 was shown to guard against thermal denaturation and radiation-induced breaks by binding to DNA *in vitro* (26, 27). Alternatively, MC1 might bend DNA to bring distal DNA loci into proximity for some regulatory purpose (28, 29). Finally, MC1 could stabilize supercoils and thus be critical for genome condensation (22–25). Since MC1 always co-occurs with a histone, it is also worth entertaining the notion that the two proteins work synergistically in some capacity (8). Here, we use the genetically tractable mesophilic methanogen, *Methanosarcina acetivorans,* to delineate the role of histones and MC1 proteins *in vivo*. Our observations are consistent with partially overlapping roles for the two classes of **D**NA-**b**inding **p**roteins (DBPs) when cells encounter stressors or during periods of quiescence.

## RESULTS

### The histone and MC1 proteins are individually dispensable in *Methanosarcina acetivorans*

**Fig 1:**
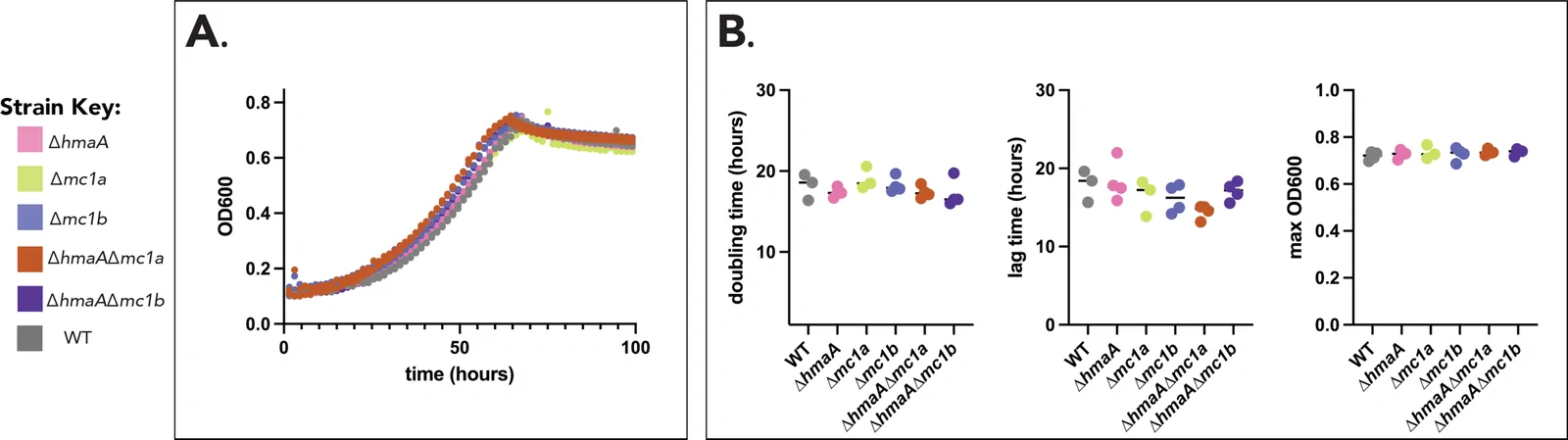
DNA-binding proteins (DBPs) are individually dispensable in *M. acetivorans*. (**A**) Automated growth curves of DBP deletion mutants (colored circles) and the parent strain (WWM60; denoted as wild type or WT; gray circles), measured as a change in optical density at 600 nm (OD600) over time in high-salt (HS) minimal medium supplemented with 50 mM trimethylamine as the growth substrate. Two representative growth curves of at least three biological replicates are shown. (**B**) Quantification of doubling time (in hours), lag time (in hours), and cell yield (measured as maximum OD600) for at least three replicates of each strain shown in panel **A**. The horizontal line represents the mean of the replicates. No significant differences from WT in doubling time, cell yield, and lag time were observed using a one-way ANOVA with Dunnett’s multiple-comparisons test. See Table 1 for quantification of growth parameters from these growth curves.

*M. acetivorans* encodes three annotated DBPs, a histone (HMaA) and two MC1 proteins (MC1a and MC1b), as monocistronic genes (Fig. S1A). Sequence- and structure-alignment suggest that these DBPs have maintained all the critical residues that are involved in binding DNA (Fig. S1). A previous study showed that the histone from *Methanosarcina mazei* Gö1 (HMm), while dispensable, is important for optimal growth (18). To the best of our knowledge, mutational studies of the *mc1* gene(s) have not been reported. Hence, we first chose to delete each DBP in *M. acetivorans* individually using our well-established CRISPR-Cas9 genome-editing methodology (30). We found that all three DBPs in *M. acetivorans* are individually dispensable (Fig. 1). Whole-genome sequencing verified that the corresponding locus was absent in each of the single-deletion mutants and, even though a few intergenic regions had low coverage, there were no mutations in any other ORFs (Table S1). *M. acetivorans* is a metabolically versatile methanogen that can grow on a variety of methylated compounds (via methylotrophic methanogenesis) as well as on acetate (via acetoclastic methanogenesis) (31). We assayed the growth of the single-knockout mutants on a panel of substrates that represent the diversity of methanogenic pathways encoded in *M. acetivorans*. None of the mutants had any observable growth defect on methanol or trimethylamine (TMA), the two methylated compounds routinely used to assay methylotrophic growth under laboratory conditions (Fig. 1A and Fig. S2 and S3A; Table 1). Only the ∆*hmaA* mutant grew 20% slower than the parent strain (WWM60; referred to as wild type or WT henceforth) on acetate (Fig. S3B). To ensure that the aforementioned growth phenotypes did not stem from changes in cell shape or size, we measured these parameters for all single knockouts and did not observe any significant differences relative to WT (Fig. S4). Taken together, each DBP is dispensable across all methanogenic conditions tested, and we did not detect any severe substrate-specific growth phenotypes for a single DBP mutant.

**TABLE 1:** Growth data for DBP deletion strains under standard laboratory growth conditions^*a*^

Strain	Substrate	Doubling time (h)	Lag time (h)	Final OD (OD600)
WT	TMA*	18.2 ± 1.6	17.9 ± 2.0	0.72 ± 0.02
∆*hmaA*		17.3 ± 0.6	18.3 ± 2.6	0.73 ± 0.02
∆*mc1a*		19.0 ± 1.4	16.4 ± 2.3	0.73 ± 0.03
∆*mc1b*		18.3 ± 1.0	16.1 ± 1.8	0.73 ± 0.03
∆*hmaA*∆*mc1a*		17.4 ± 0.8	14.5 ± 0.9	0.74 ± 0.01
∆*hmaA*∆*mc1b*		17.2 ± 1.7	17.1 ± 1.2	0.74 ± 0.01
WT	Methanol	10.0 ± 0.2	6.6 ± 0.8	1.09 ± 0.02
∆*hmaA*		10.4 ± 0.2	6.2 ± 0.3	1.11 ± 0.02
∆*mc1a*		10.6 ± 0.2	5.8 ± 0.6	1.09 ± 0.02
∆*mc1b*		10.0 ± 0.1	6.4 ± 0.4	1.10 ± 0.03
WT	Acetate	47.4 ± 3.0	127.3 ± 21.2	0.21 ± 0.01
∆*hmaA*		56.7 ± 3.2	144.8 ± 24.2	0.20 ± 0.01
∆*mc1a*		47.5 ± 2.6	131.0 ± 18.5	0.21 ± 0.00
∆*mc1b*		49.6 ± 1.7	105.5 ± 19.0	0.21 ± 0.01
WT	Acetate	49.1 ± 3.4	80.1 ± 14.9	0.16 ± 0.01
∆*hmaA*∆*mc1a*		53.8 ± 2.0	76.9 ± 4.3	0.16 ± 0.01
∆*hmaA*∆*mc1b*		55.5 ± 4.6	78.8 ± 1.5	0.16 ± 0.02

^*a*^ Doubling time, lag time, and final optical density at 600 nm (representative of growth yield) are reported for each strain under the conditions tested. All values are reported as the mean ± one standard deviation (SD). Data represent the mean of at least three replicates. Values that are significantly different from the parent strain (WWM60 denoted as wild type or WT) for a given condition (one-way ANOVA, Dunnett’s multiple comparisons test) are underlined. Substrates marked with an asterisk (*) denote growth experiments that were conducted in a 24-well microplate with an automated plate reader (see Materials and Methods). All other growth curves were conducted in anaerobic Balch tubes. Growth rates measured in 24-well microplates with a plate reader were not compared with growth rates measured in anaerobic Balch tubes.

### One copy of *mc1* is essential in *Methanosarcina acetivorans*

To test if the histone and MC1 proteins are functionally redundant, we chose to delete the DBPs in all possible combinations. We were readily able to obtain the ∆*hmaA*∆*mc1a* and ∆*hmaA*∆*mc1b* double mutants (Fig. 1A). Whole-genome sequencing revealed that these mutants have the desired deletions as well as mutations in *ade,* a gene that encodes adenine deaminase (Table S1). In previous work, we showed that null mutations in *ade* routinely occur when *M. acetivorans* is grown in medium supplemented with 8-ADP (8-aza-2,6-diaminopurine sulfate) to cure the CRISPR-editing plasmid (32). The *ade* mutations have no detectable impact on growth under laboratory conditions and can be ignored for the purpose of this work (32). Even after several attempts (as outlined in the Materials and Methods and Table S2), we were unable to obtain a double mutant that lacks both copies of *mc1* or the triple-knockout mutant. These results suggest that at least one copy of *mc1* appears to be essential in *M. acetivorans*. Next, we measured the growth parameters of the double mutants we could obtain on either TMA or acetate. Like the single mutants, none of the double mutants had any detectable growth defect on TMA (Fig. 1B; Table 1). In contrast to the ∆*hmaA* single mutant (Fig. S3B; Table 1), the ∆*hmaA*∆*mc1a* and ∆*hmaA*∆*mc1b* double mutants had no growth defect on acetate either (Fig. S5). Based on the double mutants we could construct and their growth phenotypes, we infer that the two MC1 proteins are functionally redundant for some essential role during optimal laboratory growth conditions.

### Each DBP deletion mutant exhibits a unique transcriptomic profile

To learn more about the function of each DBP in *M. acetivorans*, we obtained the global transcriptomic profile of the three single-knockout mutants and WT in minimal medium supplemented with 50 mM TMA. We chose growth on TMA for this experiment because the growth parameters of all strains were nearly identical under these conditions (Table 1), which would minimize any impact of altered growth on the transcriptome. All three DBPs were expressed in WT grown on TMA, but their expression levels varied substantially. The *mc1a* locus was expressed 3-fold more than *mc1b*, and *hmaA* was the least expressed DBP, *ca*. 3-fold lower than *mc1b* (Fig. S6). In the single-knockout background, none of the remaining DBPs changed in expression (Fig. S6), which precluded the likelihood of functional compensation at the transcriptional level. Next, we identified genes that were differentially expressed in the DBP deletion strains relative to WT. We designated a gene as being differentially expressed (DE) if it had a log_2_ fold change > +0.1 or <−0.1, i.e., ~7% change in expression, and was statistically significant (*q*-value <0.05). We chose a log_2_ fold change of 0.1 because <10 genes were differentially expressed in each strain using the typical log_2_ fold change cut-off > +1 or < -1. We reasoned that small changes in the expression of genes involved in DNA repair or regulation might have a meaningful impact on physiology and thus could be relevant for our work.

**Fig 2:**
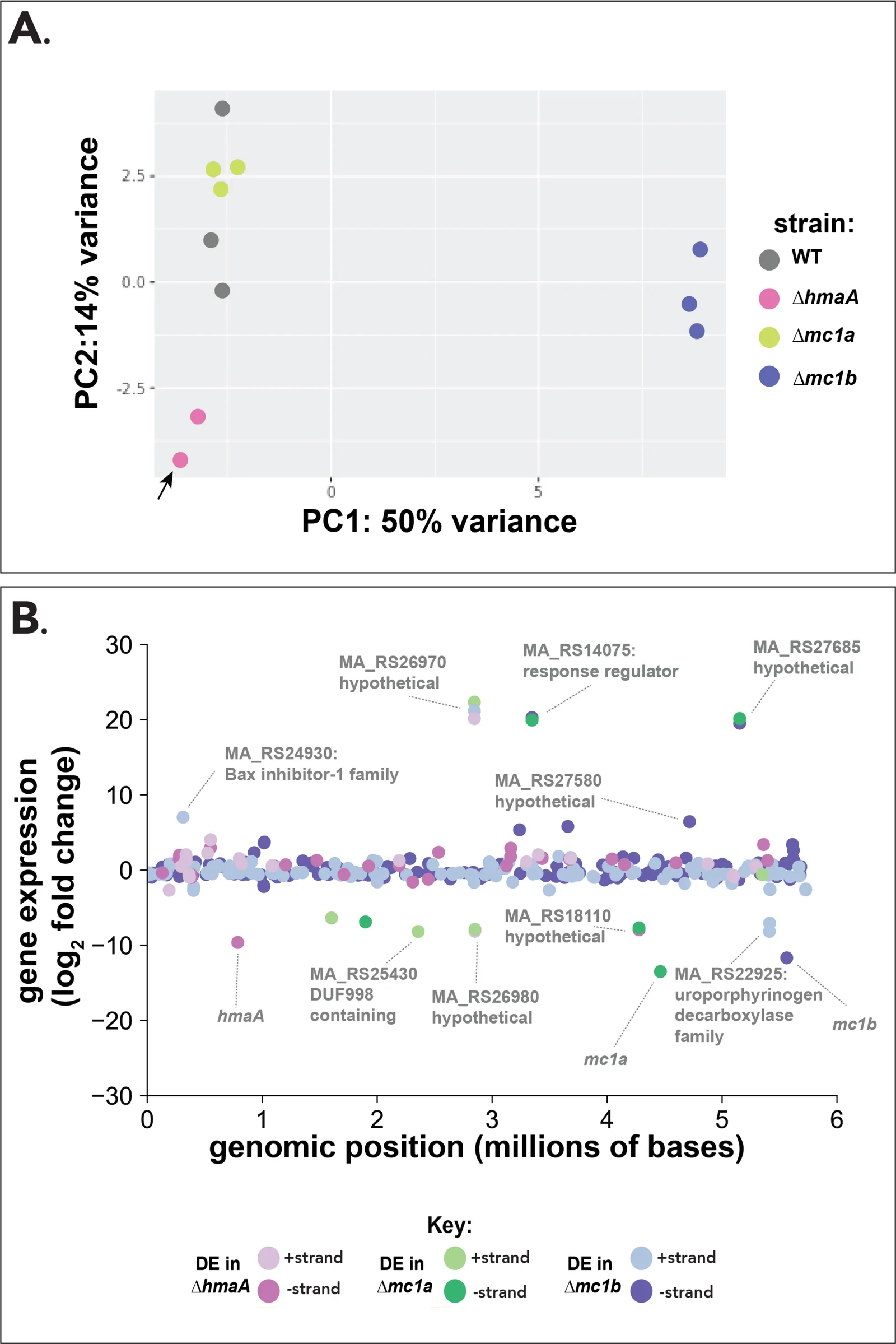
DNA-binding protein (DBP) deletion mutants have distinct transcriptional profiles compared to the parent strain. (**A**) Principal component analysis (PCA) plot showing transcriptional profiles of the three DBP single-deletion mutants (colored circles) and the parent strain (WWM60; denoted as wild type or WT; gray circles) grown in high-salt (HS) minimal medium with 50 mM trimethylamine (TMA). The black arrow indicates two points that overlap. (**B**) Scatter plot depicting genomic location and strand information for differentially expressed (DE) genes in the single-DBP deletion strains. Pink, green, and blue circles denote genes that are differentially expressed in the ∆*hmaA*, ∆*mc1a,* and ∆*mc1b* mutants. Strand information is given by the shade of the circle: light circles show genes on the positive strand, while dark circles show genes on the negative strand. Genes with a large fold change (>log_2_ 7.5-fold) in any of the mutants are labeled with locus tags and annotation information.

At a global level, each DBP single-deletion mutant exhibits a unique transcriptional profile (Fig. 2A and Table S3). Across all mutants, the DE genes did not localize to any region of the genome or to either the + or – strand (Fig. 2B). As expected, the HMaA locus was the most downregulated gene (by ~500-fold) in the ∆*hmaA* mutant (Table S3), and 60 other genes were DE in the ∆*hmaA* strain compared to WT (Fig. 2A and Table S3). Genes encoding a putative tungstate/molybdate transporter (ModABC), the molybdate-dependent formyl-methanofuran dehydrogenase (Fmd), as well as the tungstate-dependent formyl-methanofuran dehydrogenase (Fwd), were all significantly upregulated in the ∆*hmaA* mutant (Table S3). Fmd/Fwd mediate the oxidation of formyl-methanofuran (CHO-MF) to CO_2_, which is the last step in the oxidative branch of methylotrophic methanogenesis (31). The functional ramifications of modulating Fmd/Fwd transcript levels are not apparent since the growth of the ∆*hmaA* mutant is unaltered on methylated substrates such as TMA and methanol (Fig. 1 and Fig. S3A; Table 1). In addition, genes encoding transposases, components of the secretion pathway, and a hybrid sensor kinase/response regulator implicated in regulating methylotrophic methanogenesis (MA_RS22890) were also significantly upregulated in the ∆*hmaA* mutant (Table S3).

Approximately 30% of the DE genes in the ∆*hmaA* mutant were shared with at least one of the ∆*mc1* deletion mutants (Fig. 2B; Table S3). Even though MC1a is the most highly expressed NAP (Fig. S6), only 12 genes were DE in the ∆*mc1a* mutant (Table S3). In contrast, nearly 10% of the genes in the genome (405) were DE in the ∆*mc1b* mutant (Table S3). Genes belonging to all the Clusters of Orthologous Groups categories were DE in the ∆*mc1b* mutant (Table S4), but some putative regulators of methanogenesis are especially of note. Genes encoding CobW family GTP-binding proteins (MA_RS04460 and MA_RS22945) and TCP1-cpn60 chaperone family proteins (MA_RS04465 and MA_RS22935) that are found in close genomic proximity to methyltransferases required for methylotrophic methanogenesis were downregulated ~5-fold in the ∆*mc1b* mutant (Table S3). Other putative regulatory proteins encoded near methyltransferases, such as members of the hydantoinase/oxoprolinase family of proteins (MA_RS04470, MA_RS23790, and MA_RS23785), were upregulated ~3-fold in the ∆*mc1b* mutant (Table S3). Since the role of these regulatory proteins is currently unknown, their impact on the activity of neighboring enzymes and on cell physiology as a whole is not clear, especially since no tangible changes in growth parameters could be detected for the ∆*mc1b* mutant (Table 1). Two genes encoding putative smORFs with unknown functions (MA_RS26970 and MA_RS27685) and a partial CDS for a response regulator (MA_RS14075) were differentially expressed in both the ∆*mc1a* and ∆*mc1b* mutants (Fig. 2B). Thus, even though our genetic analyses suggest that the two *mc1* genes have some functional overlap, it is not necessarily evident in the global transcriptome of the ∆*mc1a* and ∆*mc1b* mutants, which are quite distinct and mostly non-overlapping.

### Stressors have a negative or positive effect on the growth of DBP mutants in *Methanosarcina acetivorans*

**Fig 3:**
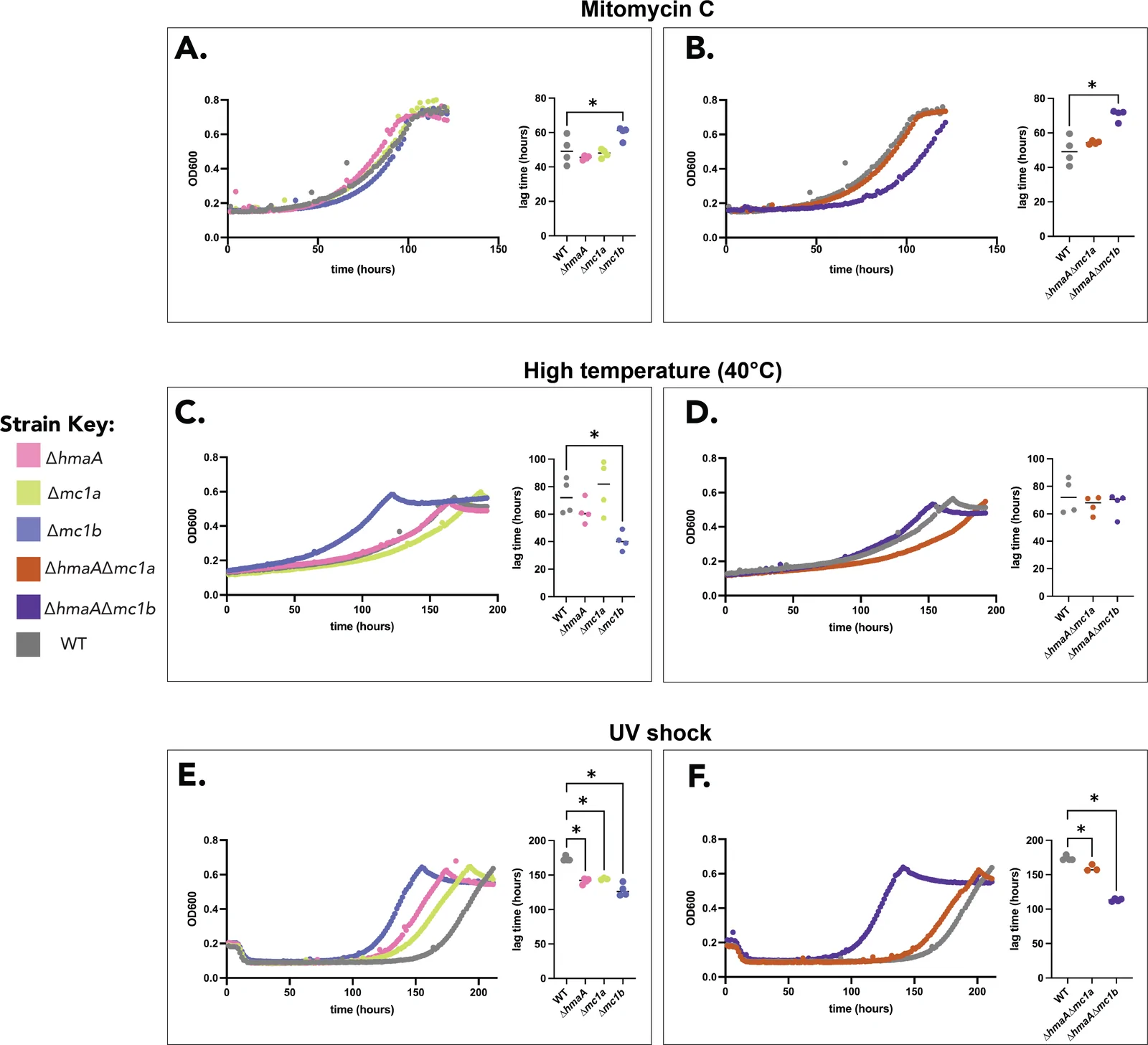
Growth of DNA-binding protein (DBP) deletion mutants after application of an abiotic stress. Growth curves of DBP deletion mutants (colored circles) and the parent strain (WWM60; denoted as wild type or WT; gray circles): (**A**) and (**B**), in medium supplemented with 1 μM mitomycin C (MMC); (**C**) and (**D**) at elevated temperature (40°C); and (**E**) and (**F**) following UV shock (see Materials and Methods) and transfer to recovery medium. Each condition is split to depict single-DBP deletion strains on the left (panels** A, C,** and** E**) and double-DBP deletion strains on the right (panels** B, D,** and** F**). Growth is measured as a change in optical density at 600 nm (OD600) over time in high-salt (HS) minimal medium supplemented with 50 mM trimethylamine as the growth substrate. A representative growth curve. from at least three biological replicates per strain is shown. The lag time (in hours) was quantified for at least three biological replicates, and the horizontal line represents the mean of the replicates. Statistically significant differences relative to WWM60 (*P* ≤ 0.05) based on a one-way ANOVA with Dunnett’s multiple-comparisons test are depicted with an asterisk. See Table S5 for quantification of the growth parameters shown in this figure.

Unlike the natural environment, laboratory conditions are optimized for fast growth and are devoid of abiotic or biotic stressors to which DBPs might specifically respond. Indeed, the histone deletion mutant of *M. mazei* Gö1 was shown to be more sensitive to UV shock than the parent strain (18). To test the role of DBPs in stress response, we measured the growth phenotype of the single- and double-DBP deletion mutants in the presence of a known DNA-damaging chemical agent (mitomycin C; MMC), at high temperature (40°C), or after UV shock. We chose these stressors because they have been known to impact the growth of histone or NAP mutants in other archaea or bacteria (16–18, 33, 34). In the presence of 1 μg/mL MMC, a few mutants had a significant growth defect. For example, the ∆*mc1b* mutant and the ∆*hmaA*∆*mc1b* mutant had 20% and 40% longer lag phases, respectively (Fig. 3A and B and Fig. S7A; Table S5). At elevated temperatures, the response was strain-specific. The ∆*hmaA*∆*mc1a* mutant grew ~40% slower, whereas the ∆*mc1b* mutant had a ~40% shorter lag time than WT (Fig. 3C and D and Fig. S7B; Table S5). The ∆*hmaA* and ∆*mc1b* single-knockout mutants recovered ~20% faster than WT after exposure to UV shock (Fig. 3E and Fig. S7C; Table S5). This growth advantage was further amplified in the ∆*hmaA*∆*mc1b* double mutant, which had a 35% shorter lag time than WT under the same conditions (Fig. 3F; Table S5). Taken together, mutants lacking DBPs have a varied response to stressors: some respond negatively to DNA-damaging agents such as MMC, whereas others respond positively to UV shock.

### Mutants lacking DBPs recover faster from stationary phase in *Methanosarcina acetivorans*

**Fig 4:**
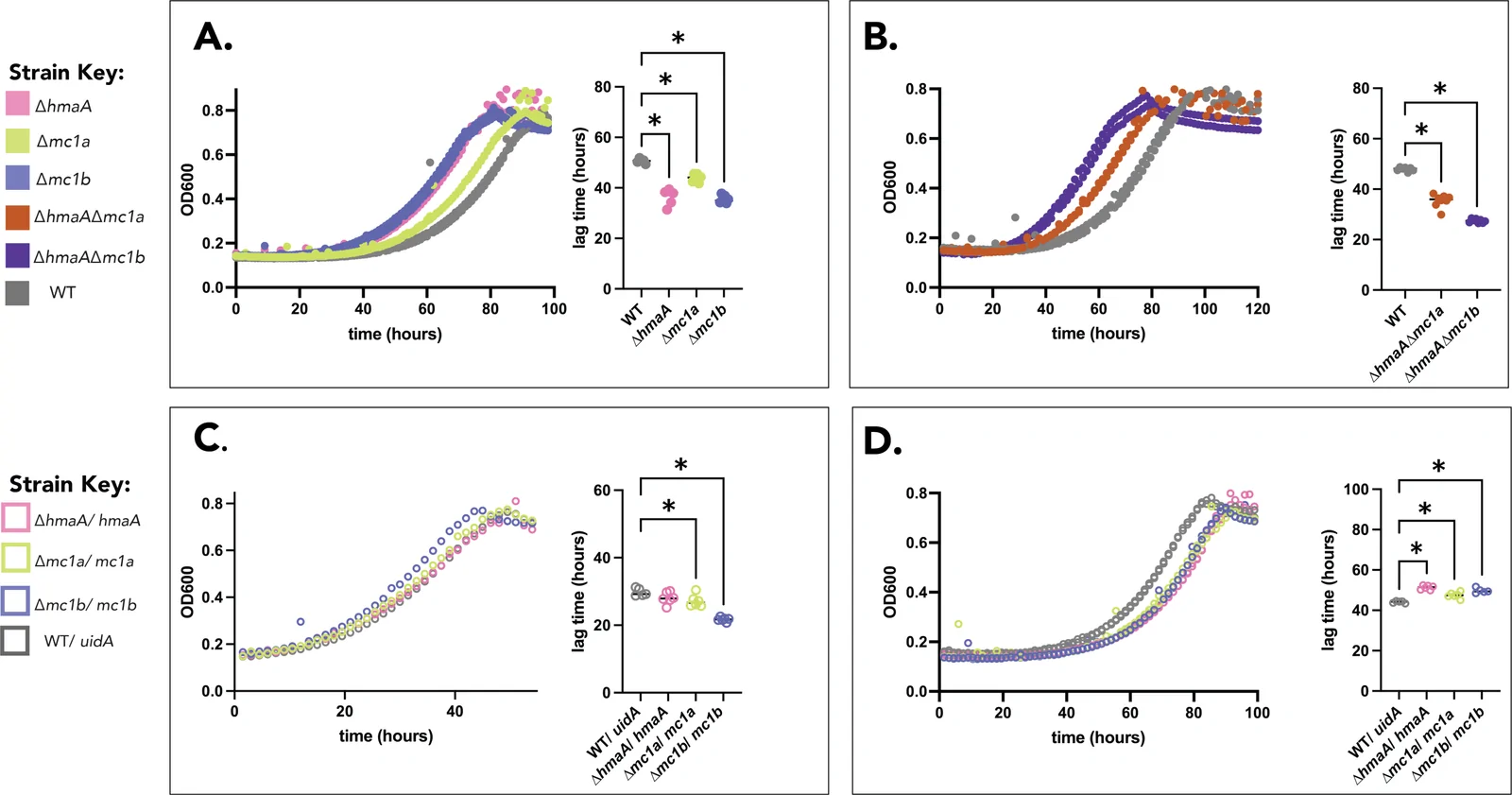
DNA-binding proteins (DBPs) affect timing of exit from extended stationary phase in *Methanosarcina acetivorans***.** (**A**) Growth curves of the single-DBP deletion mutants (colored closed circles) in comparison to the parental strain (WWM60, denoted as wild type or WT in gray closed circles), measured as a change in optical density at 600 nm (OD600). Strains were incubated for 21 days in stationary phase and inoculated into high-salt (HS) minimal medium supplemented with 50 mM trimethylamine as the growth substrate. (**B**) Growth of double-DBP deletion strains (colored circles) compared to the WT strain (gray circles) under the same conditions as panel **A**. (**C**) Growth of strains overexpressing DBPs in the respective DBP-deletion background (colored open circles) compared to the parental strain expressing an equivalent amount of *uidA* (encoding β-glucuronidase) (gray open circles). Growth curves in panel **C.** were obtained after incubation of strains for 1 day in stationary phase. (**D**) Growth curves of the same strains in panel** C** after incubation for 21 days in stationary phase. Growth medium was supplemented with 2 μg/mL puromycin and 100 μg/mL tetracycline to select for maintenance of the expression vector and to fully induce expression of the gene, respectively (see Materials and Methods). Two representative growth curves of at least three biological replicates for each strain are shown. The horizontal line represents the mean of the biological replicates. Statistically significant differences relative to the WT control strains (*P*-value ≤ 0.05), based on a one-way ANOVA with Dunnett’s multiple-comparisons test, are depicted with an asterisk. See Table S6 for quantification of the growth parameters shown in this figure.

A notable phenotype from our stress-response assays was that mutants lacking HMaA and/or MC1b could recover faster under certain conditions (Fig. 3). We postulated that this phenotype could either stem from the activation of a stress-response mechanism or an override of growth arrest caused by the DBPs. To decouple these two effects, we measured the growth of DBP deletion strains after extended incubation at stationary phase, i.e., after cells had ceased growing in batch culture, without any application of abiotic stress (see Materials and Methods for more details). After experiencing stationary phase for *ca*. 24 h, all strains behaved identically (Fig. 1B). However, after prolonged incubation at stationary phase, i.e., after 21 days, all the DBP mutants recovered substantially faster than the WT (Fig. 4A and B and Fig. S8A and B; Table S6). Notably, the ∆*hmaA* and the ∆*mc1b* single mutants had a ~30% shorter lag, whereas the ∆*hmaA*∆*mc1b* double mutant recovered even faster with a ~40% shorter lag than WT (Table S6). To test if the differences in lag time stem from changes in cell viability, we measured factor 420 (F_420_) fluorescence of WT and the mutants at various growth phases. F_420_ is a redox cofactor in methanogens that emits light in the blue-green range and is present in the cytosol of cells that have maintained intact membranes. F_420_ fluorescence is routinely used to distinguish between live and dead methanogen cells (35, 36). We observed little to no difference in the number of intact cells between WT and the DBP mutants after 21 days of incubation at stationary phase using F_420_ fluorescence as a readout (Fig. S9A and B). In contrast, the number of intact cells for WT dropped by ~70% after 72 h of exposure to puromycin, an antibiotic that inhibits translation in *M. acetivorans* (37) (Fig. S9C). These data suggest that mutants lacking DBPs indeed have a shorter lag period for growth recovery after extended periods of stasis compared to WT.

### DBPs bind DNA to modulate exit from stationary phase in *Methanosarcina acetivorans*

To test if the DBPs have direct control on growth recovery from extended stationary phase, we overexpressed each of them in *M. acetivorans*. We reasoned that overexpression of DBPs would slow down growth recovery if they directly impacted the underlying mechanism. To this end, we overexpressed each DBP on a plasmid under the control of a tetracycline-inducible promoter (37) in the respective single-deletion background. We confirmed expression by immunoblotting for a FLAG tag at the C-terminus of the protein when the cells were grown in the presence of tetracycline (Fig. S10A and B). When DBPs were overexpressed, we observed a statistically significant delay in lag time for growth recovery after 21 days of incubation in stationary phase (Fig. 4C and D and Fig. S8C and D; Table S6). The growth phenotypes of the overexpression mutants suggest that the DBPs have direct control on growth inhibition and also indicate that the presence of the FLAG tag does not impact protein function. Since the overexpression of HMaA and MC1b led to a >10% increase in lag compared to WT, we pursued these two DBPs further (Table S6).

**Fig 5:**
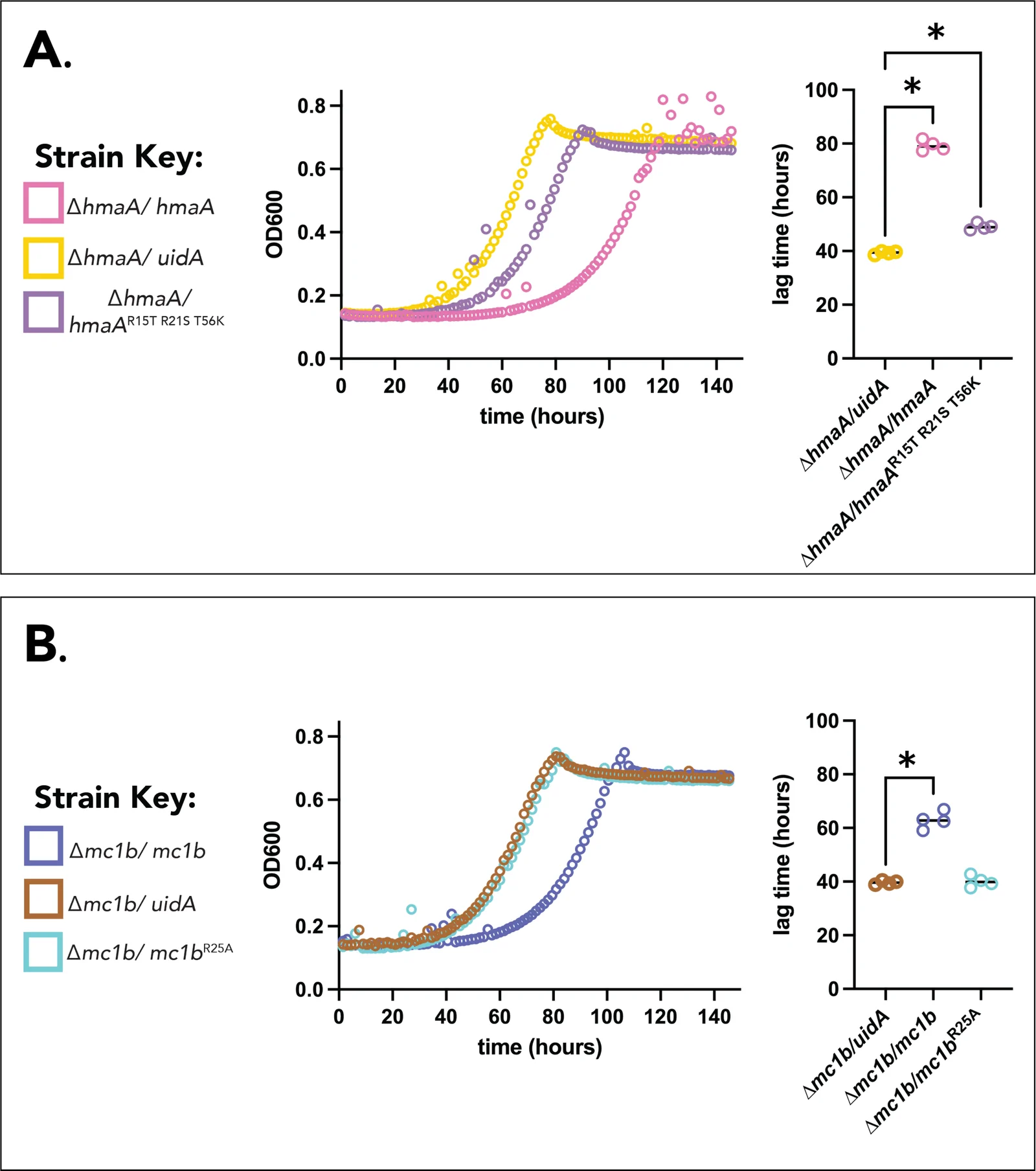
Abrogating DNA-binding residues in DBPs shortens lag time in *Methanosarcina acetivorans*. (**A**) Growth curves of ∆*hmaA-*derived strains expressing either HMaA:FLAG (pink open circles), HMaA ^R15T R21S T56K^: FLAG (lavender open circles), or UidA (yellow open circles) following 28 days in stationary phase. (**B**) Growth curves of ∆*mc1b-*derived strains expressing either Mc1b:FLAG (blue open circles), MC1b ^R25A^: FLAG (brown open circles), or UidA (cyan open circles) following 28 days incubation in stationary phase. For both panels, one representative growth curve of at least three biological replicates per strain is shown. All growth curves were conducted in high-salt (HS) minimal medium with 50 mM trimethylamine (TMA) supplemented with 2 μg/mL puromycin and 100 μg/mL tetracycline to select for maintenance of the expression vector and to fully induce expression of the target protein, respectively (see Materials and Methods). The lag time (in hours) was quantified for at least three biological replicates of each strain shown. The horizontal line represents the mean of the biological replicates. Statistically significant differences relative to the WT control strains (*P*-value ≤ 0.05), based on a one-way ANOVA with Dunnett’s multiple-comparisons test, are depicted with an asterisk. See Table S7 for quantification of the growth parameters shown in this figure.

To test if growth recovery from stationary phase is contingent on DBPs associating with the genome, we overexpressed mutant alleles of *hmaA* and *mc1b* that lack residues critical for forming a complex with DNA. Soares et al. (38) have shown that three substitutions (R13T + R19S + T54K) in the histone from *M. fervidus,* HMfB, completely abolish DNA-binding *in vitro*; hence, we generated the corresponding variant (HMaA^R15T/R21S/T56K^) for *M. acetivorans*. Paquet et al. (39) demonstrated that the R25A variant of MC1 from *M. thermophila* CHTI55 has a 100-fold higher K_d_ for DNA, so we expressed MC1^R25A^ in *M. acetivorans* to test the impact of DNA binding *in vivo*. These point mutants were FLAG-tagged at the C-terminus and expressed in the respective knockout backgrounds as described for the WT allele of each gene (Fig. 4C and D). By immunoblotting for the FLAG tag, we observed that the mutant allele of each DBP was produced at levels comparable to the wild-type allele (Fig. S10C and E). We then measured growth recovery after 28 days in stationary phase when the mutant alleles of these DBPs were overexpressed in the corresponding deletion background (Fig. 5 and Fig. S11). Overexpression of HMaA^R15T/R21S/T56K^ decreased the lag time by 40% compared to HMaA^WT^ (~50 hours versus ~80 h), but did not completely phenocopy the ∆*hmaA* deletion mutant, likely because it retains some ability to bind DNA (Fig. 5A; Table S7). In contrast, overexpression of MC1b^R25A^ phenocopied the ∆*mc1b* deletion mutant (Fig. 5B; Table S7). Taken together, these data suggest that both classes of DBPs in *M. acetivorans* likely bind the genome when cells are not actively growing.

## DISCUSSION

Even though there are only three annotated DBPs in *M. acetivorans,* all of them seem to be dispensable, which is inconsistent with a dedicated role for any one of them in genome compaction (Fig. 1 and Fig. S3 and S5). One plausible explanation is that the two MC1 proteins work in concert to condense the genome, but this seems unlikely since the expression level of MC1b is lower than that of typical genome-packaging proteins and is unaltered in the absence of MC1a (Fig. S6). Hence, it is plausible that other uncharacterized DBPs might play a role in genome compaction in *M. acetivorans*. Furthermore, none of the DBP deletion mutants impact fitness under optimal laboratory growth conditions (Table 1). These observations deviate from a previous study conducted in *M. mazei* Gö1, where growth on methanol and TMA was severely compromised (~2-fold slower) in the absence of the *hmm* locus (18). The deletion of the *hmm* locus led to global transcriptional dysregulation in *M. mazei* Gö1 and also sensitized the cells to UV shock (18). In contrast, the ∆*hmaA* mutant of *M. acetivorans* is more resilient to the same dose and duration of UV shock (Fig. 3E; Table S5). One possibility for the phenotypic differences between the two closely related strains is the mode of mutant generation. Here, we generated a clean knockout using our Cas9-based genome-editing technique; meanwhile, the corresponding mutant in *M. mazei* has a *pac* cassette, encoding puromycin *N-*acetyltransferase, under the control of the strong *mcr* promoter (P*mcrB-pac*) in place of the *hmm* locus (18). It is plausible that *pac* overexpression itself could have impacted the growth phenotype of the ∆*hmm::PmcrB-pac* mutant. Alternatively, we cannot rule out the possibility that the cellular function of the two histone proteins, even though they share ~95% amino acid identity, is variable across *Methanosarcina* species.

At least one copy of *mc1* is conserved throughout the family *Methanosarcinaceae*; however, several strains, including *M. acetivorans,* encode multiple copies (Fig. S12). Our analyses suggest that the cellular functions of the two MC1 proteins, despite being 90% identical at the amino acid level (Fig. S1), are mostly non-overlapping. MC1a is expressed ~3-fold more than MC1b (Fig. S6), and the transcriptomic profiles of the ∆*mc1a* and ∆*mc1b* mutants are very distinct (Fig. 2A), which indicates that they have undergone neofunctionalization post-duplication, likely through evolution of their regulatory elements. However, the two proteins still shared some core function that is essential (Table S2) but unrelated to genome packaging or maintenance. Similarly, Čuboňová and colleagues have also shown that the two *Thermococcus kodakarensis* histone paralogs are individually dispensable (albeit with a fitness cost) but cannot be deleted in tandem (40).

Intriguingly, many of the DBP deletion mutants in *M. acetivorans* have a growth advantage, which is contrary to the observed phenotype of related mutants in other archaea. For instance, in halophiles, deletion of histones leads to reduced fitness, even under optimal growth conditions (16). Similarly, in *Saccharolobus islandicus,* knockdown of the Alba family protein Sis10b slows down growth compared to the parental strain (41). In *Thermococcus kodakarensis,* deletion of the histone paralog HTkA abolishes transformation competency, whereas an HTkB knockout has slower growth and lower fitness (40). That said, it has been routinely shown that NAPs are dispensable in bacteria and that the knockout mutants can have a growth advantage too. For example, H-NS is not essential in *Escherichia coli* (34, 42). In fact, deletion of *hns* leads to a growth advantage at low pH and high osmolarity (34), and the *hns* point mutant *hns-66* confers a growth advantage after prolonged stationary phase (42). Similarly, *E. coli* strains lacking *fis* have increased growth rates and biomass yields in acetate minimal medium (43). Thus, it stands to reason that the fitness impacts of prokaryotic DBPs are specific to the individual organism and the growth conditions and cannot be broadly generalized.

Our finding that DBPs have growth phase-specific phenotypes is also consistent with prior work. For example, deletion of histone *hpyA* in *H. salinarum* results in a growth phase-dependent morphological phenotype (44). In *S. islandicus,* Lrs14 has a growth phase- and stress-specific expression pattern (45). Altogether, it seems likely that DBPs in archaea might play a role in protecting genomic DNA during periods of sub-optimal conditions. While the precise molecular mechanisms of DBP function under these conditions remain to be elucidated, we suggest that DBPs likely benefit the organism by promoting long-term survival, perhaps by favoring growth arrest until the organism encounters an environment that is more consistently favorable to resume growth. Indeed, as most microbes in their natural environment are not experiencing extended periods of exponential growth, this may explain why DBPs such as histone and MC1 are highly conserved across the archaeal tree (8), yet, in some organisms, are easily dispensable under laboratory growth conditions.

## MATERIALS AND METHODS

### Structure alignment of DNA-binding proteins

The *Methanothermus fervidus* HMfB crystal structure (PDB: 1a7w) or *Methanosarcina thermophila CHT155* MC1 solution structure (PDB: 2khl, model 1.1) was aligned with the AlphaFold2 (46) predicted structure for *Methanosarcina acetivorans* HMaA (AF-Q8TSX3-F1_model_v4.pdb, AlphaFold Protein Structure Database) and *M. acetivorans* MC1a (AF-Q8TK06-F1-model_v4.pdb, AlphaFold Protein Structure Database) or *M. acetivorans* MC1b (AF-Q8THJ8-F1_model_v4.pdb, Alpha Fold Protein Structure Database), respectively, using the matchmaker command in UCSF ChimeraX (47) (version 1.7.1). The root mean square displacement output is reported based on the pruned structure. For the HMfB-HMaA alignment, the pruned structure represents 66 of 67 possible atoms. For the MC1-MC1a alignment, the pruned structure represents 71 of 93 possible atoms. For the MC1-MC1b alignment, the pruned structure represents 72 of 93 possible atoms.

### Plasmid construction

Plasmids for CRISPR-Cas9-mediated genome editing were cloned as previously described (26). Single-guide (sg)RNAs were designed using the CRISPR site tool in Geneious Prime (version 2024.0.5). PCR fragments containing the sgRNA scaffold were assembled into pDN201 linearized with *AscI* as previously described (30). The appropriate homologous repair template was assembled into the sgRNA-containing derivative of pDN201 at the *PmeI* site. For complementation plasmids, PCR fragments containing genes of interest were amplified from the *M. acetivorans* genome or from commercially synthesized gene fragments (gblocks) containing the desired point mutations, as applicable. When applicable, epitope tags were added to gene fragments using a primer containing the desired tag sequence. Primer information can be found in Table S8. PCR fragments containing genes of interest and tags, when appropriate, were fused to the appropriate tetracycline-inducible promoter by linearizing either pJK027A or pJK029A with *NdeI* and *HindIII*. Plasmids were verified by Sanger sequencing. For expression in *M. acetivorans,* pJK027A/pJK029A derivative plasmids were co-integrated with pAMG40. Co-integration was conducted using the Gateway BP Clonase II Enzyme Mix and verified by a diagnostic restriction endonuclease digest. All cloning was performed in WWM4489, an *E. coli* DH10B derivative, which was transformed via electroporation at 1.8 kV using an *E. coli* Gene Pulser. *Escherichia coli* strains were cultivated at 37°C with shaking at 250 rpm in lysogeny broth supplemented with 20 µg/mL chloramphenicol and/or 25 µg/mL kanamycin. *E. coli* strains carrying the co-integrated vector were cultured with 10 mM rhamnose to induce copy number as previously described (48). All primers and plasmids used in this work are cataloged in Tables S8 and S9, respectively.

### Generation of *M. acetivorans* deletion mutants

Transformation of *M. acetivorans* was conducted as described previously (49). In brief, 20 mL cultures of *M. acetivorans* were grown in high-salt (HS) minimal-medium supplemented with 50 mM TMA (HS-TMA) as the growth substrate. Transformants were plated on solidified HS-TMA + 1.5% agar medium supplemented with 2 µg/mL puromycin as a selection agent. Plates were incubated at 37°C in an anaerobic incubator with H_2_S/CO_2_/N_2_ (1,000 ppm/20%/balance) in the headspace. For complementation strains, colonies were screened by PCR and sequenced by Sanger sequencing to verify the presence of the desired plasmid construct. For deletion strains, individual colonies were picked into liquid medium, grown to saturation, and then screened via PCR at the chromosomal locus for the desired mutation. Screening primers are listed in Table S8. Colonies that were identified as having the desired deletion were then plated on solidified HS-TMA + 1.5% agar medium supplemented with 20 µg/mL 8-ADP to cure the plasmid. Single colonies were then picked and screened via PCR to confirm the absence of the *pac* (puromycin resistance) gene on the mutagenic plasmid (see Table S8 for primers). A list of strains used in this study is provided in Table S10. In the case that a desired DBP mutant was unable to be recovered (e.g., ∆*mc1a*∆*mc1b*), the transformation of the editing plasmid was repeated at least two more times, and colonies were screened as described above. In parallel, within the same background where deletion of the DBP was attempted, an editing plasmid targeting a neutral locus (MA2520, orotate phosphoribosyltransferase-like protein) was also transformed. Colonies were then screened for deletion of the neutral locus. A mutant was considered unable to be generated when deletion of the neutral locus, but not the desired DBP locus, was observed in multiple biological replicates. A summary of these experiments can be found in Table S2.

### DNA extraction and whole-genome re-sequencing

10 mL cultures of the desired strain were grown to saturation in HS minimal medium with 50 mM TMA, and DNA was extracted and processed as previously described (32). Genomic DNA was sequenced at the SeqCenter (Pittsburgh, PA) via Illumina paired-end reads. The resulting sequencing reads were subsequently mapped to the *M. acetivorans* reference genome using Breseq (50), version 0.35.5 (default parameters). In strains cured of the editing plasmid, *M. acetivorans* C2A was used as the reference genome. All mutations identified via this analysis are reported in Table S1.

### Growth assays in Balch tubes on methanol and acetate

Growth curves in Balch tubes, as delineated in Table 1, were performed by pre-culturing strains in 10 mL HS medium and the growth substrate (125 mM methanol or 40 mM sodium acetate) at 37°C without shaking in sealed Balch tubes with N_2_/CO_2_ (80/20) in the headspace at 8–10 psi. For growth experiments, Balch tubes containing sterile medium were inoculated with 0.5 mL of the pre-culture at late exponential phase (OD600 ~0.6–0.9). Growth of four independent biological replicates was monitored for each strain by monitoring the change over time in optical density of cultures at 600 nm using a UV-Vis spectrophotometer (Genesys 50, Thermo Fisher Scientific, Waltham, MA, USA). Readings were “blanked” with an uninoculated, sterile tube containing 10 mL of the corresponding medium. Growth rates were determined by finding the slope of the best linear fit of the log-transformed OD600-versus-time plot. The “best linear fit” was determined by selecting the linear model with the maximal R^2^ value (minimum cut-off ≥0.98) with a minimum of four points. Doubling times were calculated from growth rates by dividing the natural log of 2 by the growth rate. Lag time was calculated by solving the best linear fit model for time x when y was set equal to the log-transformed initial OD600 observed at the beginning of the experiment.

### Growth assays on trimethylamine (TMA) in a high-throughput microplate reader

Only growth curves with 50 mM TMA as the growth substrate were conducted in a high-throughput microplate reader format because methanol was too volatile and growth on acetate was too slow for robust optical density measurements in a high-throughput microplate reader format. Growth curves conducted in a high-throughput microplate reader format were performed using a modified growth medium that was buffered by 50 mM 1,4-piperazinediethanesulfonic acid (PIPES) adjusted to a final pH of 6.8 (HS-PIPES). All other components of the medium remained the same as described previously for bicarbonate-buffered HS medium. Strains were pre-cultured until they reached stationary phase in 10 mL HS-PIPES + 50 mM TMA in hermetically sealed Balch tubes with pure N_2_ in the headspace at 8–10 psi. Within 24 h of reaching stationary phase (unless otherwise specified), 1 mL of pre-culture was then inoculated into Balch tubes containing HS-PIPES + 50 mM TMA medium and brought into an anaerobic chamber (Coy Laboratory Products, Grass Lake, MI, USA). The contents of each Balch tube were then mixed by gently pipetting up and down with a 10 mL pipette at least 10 times to ensure homogeneous distribution before dispensing 1.8 mL into each well of a sterile, anaerobic 24-well flat-bottomed tissue culture microplate (cat. #353047, Corning Life Sciences, Durham, NC, USA). For growth curves with multiple strains, strains were assigned well locations via a random number generator to mitigate any edge effects or global position bias in the growth of an individual strain. In any given growth curve, at least four replicate wells were used for a given strain. Plates were tightly sealed with optical film (cat. #2921-7800, USA Scientific, Ocala, FL, USA) and then incubated in a microplate reader (Agilent BioTek Epoch 2, Agilent, Santa Clara, CA, USA) at 37°C. The microplate reader was programmed as follows: experiment type—24-well kinetic assay; kinetic activity—shake before each read at 425 cpm (3mm, speed: fast); primary wavelength-—600 nm. For growth curves where readings were taken every hour, the kinetic interval was set to 1 h and the shake interval was set to 59 min. For growth curves where readings were taken every 1.5 h, the kinetic interval was set to 1 h, 30 min, and the shake interval was set to 1 h, 29 min. Growth was analyzed using a MATLAB script (https://github.com/alienorbask/growthcurve). The “best linear fit” was determined by selecting the linear model with the maximal R^2^ value (minimum cut-off ≥0.99) with a minimum of eight points. Doubling time and lag time were then calculated as described above. Upon conclusion of the growth curve, if defects were observed in the seal integrity around a given well, data from that well were not considered in the analysis. For growth experiments with strains bearing a complementation plasmid in *trans*, the growth medium was supplemented with 2 µg/mL puromycin for plasmid maintenance and 100 µg/mL tetracycline for full induction of the inducible promoter.

### Growth assays with stressors in a high-throughput microplate reader

For assessment of growth with stressors, strains were cultivated as described for high-throughput microplate reader growth assays, with the following exceptions: for growth with mitomycin C, growth medium was supplemented with 1 µg/mL mitomycin C (Sigma-Aldritch, cat. # M4287). For growth at high temperature, strains were pre-acclimated by culturing in HS-TMA-PIPES in Balch tubes submerged in a 40°C water bath for three passages prior to inoculation and growth in the microplate reader at 40°C. For UV shock, strains were pre-cultured in HS-TMA-PIPES in Balch tubes as described above, then exposed to UV light as per the methods previously described (18). Briefly, exponentially growing strains (OD600 = 0.6–0.9) were brought into the anaerobic chamber and decapped, and the 10 mL pre-culture volume was dispensed into open, anaerobic petri dishes. The suspended cultures were gently stirred, then subjected to 253 nm UV light from a handheld UV lamp (iClevr, Newark, CA, USA) at 20 cm for 3 min. Following irradiation, cultures were gently stirred to ensure homogenous mixing of cells throughout the culture, and then a 3 mL volume, normalized to account for any differences in OD between strains, was inoculated into 10 mL fresh HS-TMA-PIPES medium. Recovery and subsequent growth were then monitored in 24-well plates as described above.

### Assessment of strain growth following extended stationary-phase incubation in a high-throughput microplate reader

For experiments examining stationary-phase exit, strains were pre-cultured in HS-TMA-PIPES in Balch tubes as described above. During pre-culturing, OD600 was monitored to ensure that strains were synchronized in terms of growth phase. Once strains reached saturation and entered stationary phase, as defined by the point at which OD600 stopped monotonically increasing, strains were continuously incubated at 37°C for the amount of time specified. After incubation in stationary phase, 1 mL of each pre-cultured strain, normalized to account for any differences in OD600 between strains, was inoculated into fresh HS-TMA-PIPES medium, and subsequent recovery and growth were monitored via microplate reader growth curves as described above. For strains bearing complementation plasmids, both the pre-culture medium and the medium for the growth curve experiment were supplemented with 2 µg/mL puromycin for plasmid maintenance and 100 µg/mL tetracycline for full induction of the inducible promoter. During pre-culture, Balch tubes containing medium with tetracycline were wrapped in foil for protection from ambient light.

### Electron microscopy and cell morphology analysis

Cells were fixed in 4% paraformaldehyde for 25 min, then washed once in 3× phosphate-buffered saline. Cells were then settled onto plasma-treated coverslips, post-fixed with osmium tetroxide for 1 h, then dehydrated with ethanol for 30 min. Samples were then placed in the critical point dryer for at least 2 h, sputter-coated with platinum for 30 min, and imaged on the scanning electron microscope (Phenom Pharos, Nanoscience Instruments Inc.). Since *M. acetivorans* has an irregular spherical cell morphology, we used cell width to estimate the diameter of the cells. Cell width was measured in FIJI (51) by drawing a horizontal line from edge to edge of each cell and using the “Measure” function to determine width in microns for 100 individual cells per strain. Cells were excluded from measurement if they were clumped with other cells or displayed visible extrudate suggesting that membrane integrity had been compromised.

### RNA extraction, sequencing, and analysis

10 mL cultures of the parental strain (‘WWM60’) and DBP single-knockout strains (∆*hmaA*, ∆*mc1a*, and ∆*mc1b*) were grown in quadruplicate in hermetically sealed Balch tubes with HS minimal medium and 50 mM TMA at 37°C. When cultures reached mid-exponential phase (OD600 of 0.5–0.6), 1 mL of culture was removed and RNA was extracted as described previously (32). Samples were sent to SeqCenter (Pittsburgh, PA) for Illumina paired-end sequencing. Transcriptome analysis was performed using the KBase bioinformatics platform as previously described (52). Briefly, raw reads were aligned to the *M. acetivorans* C2A reference genome using Bowtie2 (v.2.3.2); aligned reads were assembled using Cufflinks (v.2.2.1), and fold change, significance values, and a principal component analysis were calculated using DESeq2 (v.1.20.0). Default parameters were used for all programs. We designated a gene as DE from the WT strain if it exceeded a log_2_ fold change of ± 0.1 and a *q*-value of ≤0.05. DESeq2 data for all mutant strains, with respect to the WT strain, are listed in Table S3. Raw transcript reads have been deposited in the Sequencing Read Archive (SRA), project number PRJNA1365308.

### Microscopy assay to measure cell viability

1 mL of culture for a given strain at a given growth phase was spun and resuspended in 20 µL medium. 5 µL of this resuspension was mounted onto a glass slide for imaging using a Zeiss AxioImager M2 microscope. Bright-field images were captured using differential interference contrast (DIC), and factor F_420_ autofluorescence was captured in the DAPI channel. At least three random fields comprising at least 250 cells were imaged for each strain under each condition. Cells visible in each channel were quantified in FIJI for each image as follows: image thresholds were adjusted to select cells and then converted to binary. The “Binary > Fill Holes” and “Binary > Watershed” commands were run to ensure selection of individual cells. The “Analyze > Analyze Particles” command was run with the size selection from 10 to infinity; otherwise, default parameters were used. The cell count was determined by the “Count” output. The percentage of intact cells was determined by dividing the count of cells in the DIC channel by the count of cells in the DAPI channel for each field of cells.

### Immunoblotting of FLAG-tagged proteins

Strains bearing overexpression plasmids for FLAG-tagged proteins were grown to saturation in medium with 2 µg/mL puromycin (to maintain the plasmid). When appropriate, 100 µg/mL tetracycline was added for full induction of gene expression. Cell lysates were prepared and processed as described in detail previously by our lab for *M. acetivorans* mutants (52). Total protein concentration in cell lysates was quantified using Bradford reagent (Sigma-Aldrich, St. Louis, MO) with bovine serum albumin standards, per the manufacturer’s instructions. Volumes of cell lysate containing approximately equal quantities of protein were mixed with 2× Lammeli loading buffer (β-mercaptoethanol added), then loaded onto a 4%–20% gradient Mini-PROTEAN TGX gel (Bio-Rad). Following separation on the gel, proteins were transferred to a Trans-Blot Turbo Mini 0.2 µM PVDF membrane using the Bio-Rad Trans-Blot Turbo Transfer System. FLAG-tagged proteins were visualized using monoclonal anti-FLAG M2-Peroxidase (HRP) antibody (Sigma-Aldrich, St. Louis, MO) at a 1/30,000 dilution. Signal from the antibody was detected using Immobilon Western Chemiluminescent HRP Substrate (Millipore, Burlington, MA) with images collected via a ChemiDoc MP Imaging System (Bio-Rad, Hercules, CA). Exposure time for each blot was 1 or 3 s, as indicated in Fig. S10. For panel E of Fig. S10, a clean paper towel was placed over the right half of the blot to mask the bright bands present on that side of the blot and allow for better visualization of the dimmer bands on the left side of the blot. The same blot, with no mask, is shown in Fig. S10D with an exposure time of 1 s. As a loading control for each Western blot, duplicate samples were loaded onto a second gel of the same composition and layout; this gel was stained and protein bands were visualized using GelCode Blue (ThermoFisher, cat. #24590). Loading controls are depicted in Fig. S10A through C.

### Bioinformatic screen for histone and *mc1* genes within the *Methanosarcinaceae*

Archaeal genomes from GTDB r.214.0 were downloaded, annotated, and checked for genome completeness as previously described (32). Profile HMMs for the archaeal histone (accession: NF043032.1) and MC1 (accession: PF05854.hmm) were obtained from NCBI and InterPro, respectively. The HMM Gene Search Tools were used with default parameters. All protein hits were counted, and the number of hits was recorded for each genome. This information was overlaid on a tree of *Methanosarcinaceae* from the GTDB ar53_r214.tree using iTOL (53).

## Supplementary Material

Reviewer comments

## Data Availability

Sequence reads for whole genome sequencing and RNA sequencing in this study have been uploaded to the Sequencing Reads Archive (SRA); project number PRJNA1365308. Custom scripts used for growth measurement can be found at https://github.com/alienorbask/growthcurve.

## SUPPLEMENTAL MATERIAL

Supplemental figures spectrum.02785-26-s0001.pdf Fig. S1 to S12.

Supplemental tables spectrum.02785-26-s0002.xlsx Tables S1 to S10.

OPEN PEER REVIEW reviewer-comments.pdf An accounting of the reviewer comments and feedback.

ASM does not own the copyrights to Supplemental Material that may be linked to, or accessed through, an article. The authors have granted ASM a non-exclusive, world-wide license to publish the Supplemental Material files. Please contact the corresponding author directly for reuse.
