# Supplementary material for: Histones and nucleoid-associated proteins in Methanosarcina acetivorans mediate recovery from stress and stasis
Source: Microbiol Spectr. 2026 Oct 14;14(11):e02785-26. doi: 10.1128/spectrum.02785-26 (PMC13729102; doi:10.1128/spectrum.02785-26)
Supplement: Supplementary file 1 [file reviewer-comments.pdf]

ASM | Microbiology Spectrum

Histones and nucleoid-associated proteins in *Methanosarcina acetivorans* mediate recovery from stress and stasis

Alienor Baskevitch and Dipti Nayak

Corresponding Author(s): Dipti Nayak, University of California Berkeley

Review Timeline:

Submission Date:	August 31, 2026
Editorial Decision:	September 4, 2026
Revision Received:	September 8, 2026
Accepted:	September 10, 2026

Editor: Blaire Steven

Reviewer(s): The reviewers have opted to remain anonymous.

Transaction Report:

(Note: With the exception of the correction of typographical or spelling errors that could be a source of ambiguity, letters and reports are not edited. The original formatting of letters and referee reports may not be reflected in this compilation.)

DOI: https://doi.org/10.1128/spectrum.02785-26

Re: Spectrum02785-26 (**Histones and nucleoid-associated proteins in *Methanosarcina acetivorans* mediate recovery from stress and stasis**)

Dear Prof. Dipti D Nayak:

After having read the manuscript and the response to previous review, I feel that the authors have addressed all of the previous review concerns.

Thank you for the privilege of reviewing your work. I am pleased to inform you that your manuscript has been editorially accepted for publication. However, there are a few additional questions in the submission form that need to be answered before your manuscript is officially accepted. Once these are completed, please return your submission so that I can move your paper forward to acceptance.

Please return the manuscript within 60 days; if you cannot complete the submission questions within this time period, please contact me.

Thank you for submitting your paper to Spectrum.

Sincerely,
Blaire Steven
Editor
Microbiology Spectrum

Please find our response to the reviewer comments in red below. All line numbers correspond to the revised manuscript (without track changes).

Reviewer #1 comments

Summary: This manuscript addressed an important and timely topic in archaeal chromatin biology, investigating how a histone (HmaA) and two copies of an archaeal nucleoid-associated protein (Mc1a and Mc1b) contribute to genome organization, stress resilience, and growth dynamics in Methanosarcina acetivorans. To my knowledge, this is one of the very few studies that examines a histone and a NAP in combination within a single archaeal organism, which is a valuable and novel direction for understanding the broader functional diversity of archaeal DNA-binding proteins (DBPs). The unified framing of histones and NAPs as DBPs is conceptually strong and aligns with growing appreciation that classical "structural" chromatin proteins may also provide stress-responsive or regulatory roles in archaeal systems. The authors report that individual DBPs are dispensable under optimal conditions in M. acetivorans, with both Mc1 copies required for growth, but that deletion mutants recover faster after stress or prolonged stationary phase, whereas overexpression delays recovery. These findings imply partially overlapping, protective roles and add to a small but growing body of literature that challenges the long-standing notion of a universal genome-compaction function for archaeal histones. This study has strong potential, but several issues with data presentation, analysis, and interpretation need to be clarified and corrected before the conclusions can be fully supported. These issues are enumerated below and are intended to improve and clarify the study's presentation of the findings.

Major Concerns

Additional background and context are needed to fully develop the rationale for mechanistic hypotheses:

- The introduction lacks essential prior information on Mc1 proteins (see papers below). Mc1 has been extensively characterized in Methanosarcina thermophila as a DNA-bending protein that protects DNA from thermal denaturation, demonstrating a stress-responsive role directly relevant to this study.
- Additional phylogenetic and structural information would strengthen the motivation for studying Mc1 in M acetivorans. To ameliorate this concern, we suggest:

o The introduction and/or first section of the results should emphasize that Mc1 has a restricted phylogenetic distribution, primarily in Methanosarcina and haloarchaea. The literature suggests Mc1 may have evolved specialized functions in these lineages that may have been selected for in extremophilic environments.

We thank the reviewer for their positive feedback. To address this specific concern (which was also raised by the other two reviewers as well) we have rewritten the introduction in the revised manuscript. The revised introduction provides a more comprehensive review of MC1 and its putative role in the context of methanogens.

To specifically address the reviewer's comment re: the restricted phylogenetic distribution and the specialized function of MC1 in the context of extremophilic environments, we do not believe these statements are accurate. A recent survey of NAPs in archaea shows that MC1 is quite broadly distributed

across archaea that are found a wide range of environments (see panels b, c from Figure 1 of a landmark paper from the group of Tobias Wernecke below).

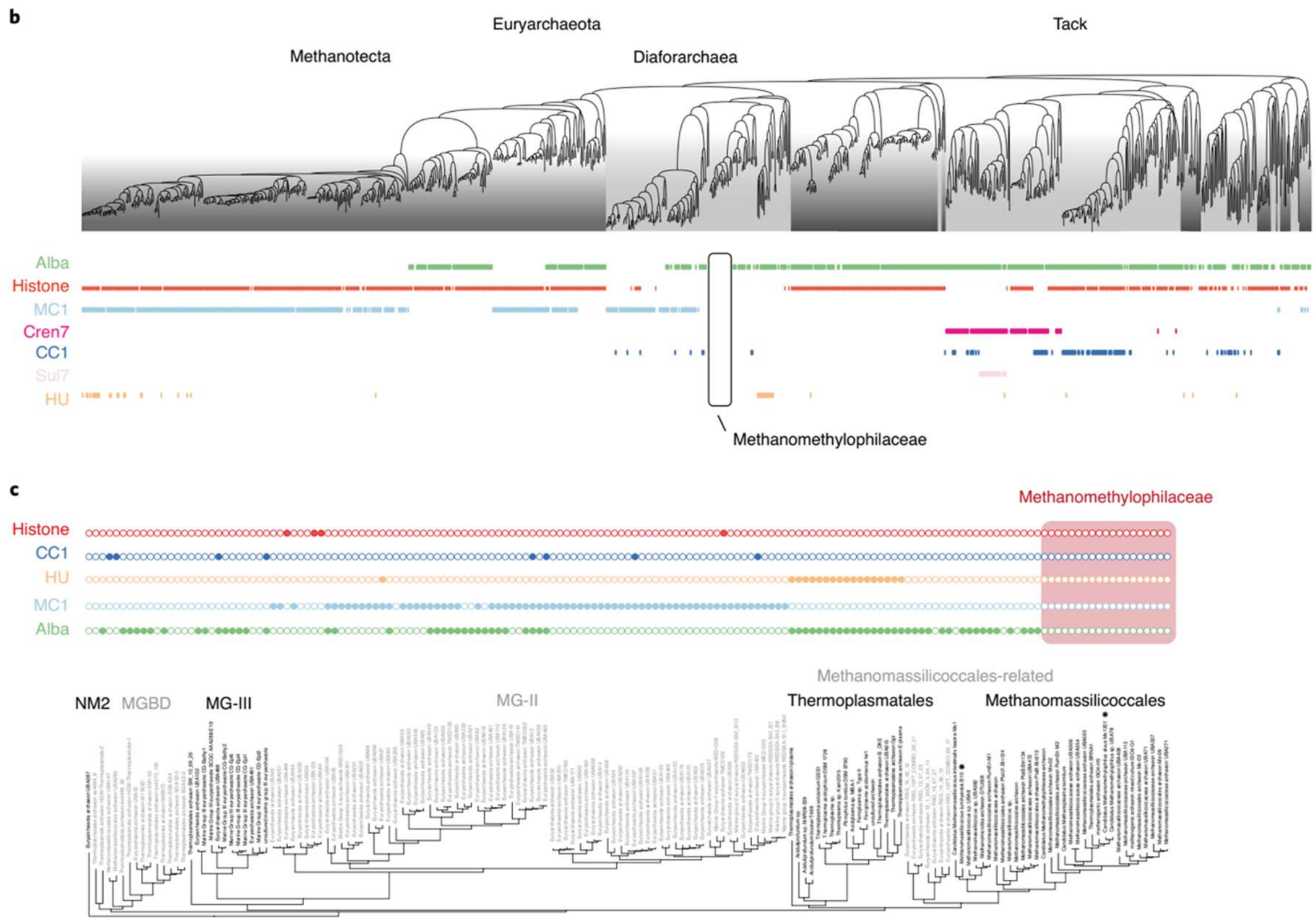

o Bringing supplementary Figure S1 into the main text (with additional explanatory text in its own results section).

We thank the reviewer for this suggestion. However, the data shown in Figure S1 are not "new results" and some of the other reviewers raised the issue that we have too many figures/supplementary materials. Hence, we have chosen to not move this figure into the main text. Instead, we have rewritten the whole introduction to provide more background information on MC1 and histones in the context of methanogens.

o Adding additional background from the literature about the known and/or expected DNA- binding modes, domain architectures, or predicted functions of the histone and Mc1 proteins. (Can the authors add information for Mc1 from Loth et al., 2019; Paquet et al., 2014; De Vuyst et al., 2005; Cam et al., 1999; Chartier et al., 1988?)

Thank you, we have revised the introduction to provide additional information as requested by the reviewer and added all of these references in the revised manuscript as well.

• Providing basic expression or proteomic abundance data for HmaA and Mc1 in M. acetivorans is necessary and would help the reader anticipate their relative biological contributions in the Discussion section on their roles.

We have provided expression data for HmaA, MC1a and MC1b in the supplement (see Supplementary Figure S6) and have discussed this in the main text (see lines 122-126 in the main text)

o The authors should report qRT-PCR data and/or summarize expression levels from existing or previously published RNA-seq datasets.

Please see our response to comment above.

o This information is important for evaluating their potential in vivo roles, as NAPs and DNA- compacting proteins are typically highly expressed, whereas proteins with primarily regulatory functions are often present at lower abundance.

Please see our response to comment above. We agree with the reviewer's assessment here. We have provided these data and discussed then in the results. Since our work extends beyond using abundance values to predict gene function, we did not want to rely on this metric to make any functional assignments per se.

RNA-seq analysis presentation needs to be clarified in order to provide further support for current claims: Overall, the RNA-seq dataset is potentially strong but could be more effectively showcased as follows:

- Several key statements about the number of DE genes in each strain need visualization to help the reader follow along with the logic and to ensure that the statements made are supported by figures or tables (e.g. "61 genes were DE in ΔhmaA, 30% overall with Δmc1 mutants" line 122-123). Please add relevant visualizations (for example, heatmaps, Venn diagrams, volcano plots) and supporting text for each mutant.

We agree with the reviewer's assessment here and have rewritten the results section to clarify key details re: our transcriptomics experiments. We have shown all the genes that were differentially expressed in each mutant in Figure 2B. We prefer the visual shown in Figure 2B to a heat map or a Venn diagram because it has more information. It shows the fold change (not captured in a Venn diagram), the overlap of differentially expressed genes (hard to parse in a heat map) and a chromosomal context (not captured in any of the options suggested above). We hope the reviewer sees our perspective and appreciates all the changes we have made to the text to address their comments.

- As the authors state, the Δmc1a strain has only 12 genes different than wild type. Because the PCA plot shows that the mutation of mc1A and hmaA explains less than 14% of the variance relative to wild type (with replicates of mc1A transcriptomes completely overlapping with wild type), our interpretation of the data is that only the Δmc1b strain has a distinct transcriptome profile relative to wild type. Therefore, please soften and add nuance to the conclusion that "each DBP has a unique transcriptional profile" (line 116).

We do not agree with the reviewer's assessment here. The PCA plot captures more nuance than just the number of genes that are differentially expressed between two mutants so the difference cannot just be attributed this one criterion. We have clarified this in the text (see lines 127-133).

- Figure 2B is difficult to interpret due to the inclusion of +/- strand labels and unclear graphical design. Simplifying by excluding the strand information would improve readability.

We think the strand information is relevant because in previous work it has been shown that NAPs might bind specifically to one strand or in certain regions of the chromosome. See https://link.springer.com/article/10.1007/s12064-020-00323-5 and https://journals.asm.org/doi/full/10.1128/jb.00119-22 as examples of the former and latter respectively. Hence, we think this is useful information relevant for people in the field and have kept it as is. We have clarified some of the details pointed out by the reviewer in the figure legend (see lines 701-711).

- The motif enrichment analysis is underdeveloped. To clarify this, the manuscript needs to explain why motif discovery matters to the central questions (for example, adding to the introduction or this section of the results), or whether these potential motifs align with known DBP/DNA-distortion biology (see comments above about structural context). Given that many NAPs may or may not bind DNA structure-dependently and sequence-specific, the biological meaning of motif presence/absence should be clarified.

We agree with the reviewer's assessment here. We have removed the entire section re: motif enrichment from the revised manuscript.

- The authors imply transcription-factor-like roles and potential genome-compaction functions for Mc1b. With only RNA-seq from one condition and growth curves, these conclusions are premature. Please soften the conclusions around these statements.

We have softened these claims throughout the text and removed any specific functional implications. We thank the reviewer for this incisive comment.

- Deletion of the predicted DNA binding residues warrants the careful claim that "DBPs...might bind DNA...", but more solid claims about binding and functional DNA-engagement should ideally be supported by EMSA, FP, ChIP-seq, or chromatin accessibility assays.

We agree but some of this work is beyond the scope of this specific paper. To address the reviewer's comment, we have tempered any claims re: DNA binding substantially throughout the text.

- Without stronger mechanistic evidence, conclusions regarding distinct functional specialization, protective genome roles, or stress-dependent chromatin regulation are overstated.

Please see our response to the comment above.

- To address these concerns, the authors should temper the strength of their conclusions and explicitly distinguish between results directly supported by the current data and more speculative models that will require further biochemical or chromatin-level validation.

Please see our response to the comment above.

Minor Concerns

Growth curve presentation and quantification

- All growth curves should be plotted on a log scale, per standard microbiological practice.

We do not agree with the reviewer's assessment here. The key parameter of the growth curve that changes in the mutants is the lag time. When you plot growth curves on a log scale it obfuscates the lag time because the initial doubling (or two) cannot be distinguished from lag. To allow the readers to "see" the changes in lag time we have kept all the growth curves as is. We hope the reviewer agrees with our assessment here. Also, we want to clarify that there are no "standard" practices, the end must justify the means. The expectation that cells will always follow a perfectly exponential growth curve is not a common observation. We often observe diauxie, linear growth etc. even under standard laboratory growth conditions.

- "Representative" curves should be explicitly defined. Mean of biological replicates vs a single biological replicate or technical replicate.

By representative we mean one of three biological replicates. We have clarified this throughout the figure legends. Thank you for point this out.

o Best practice would be mean curves or fitted curves with shaded error rather than overlapping plots in supplements.

We do not agree with the reviewer's assessment here. Each growth curve is an independent experiment and will produce a certain set of growth parameters. The statistically sound way of treating these data is to take the average of the growth parameters rather than average the raw data (i.e. OD values) for the growth curves to produce a "mean" curve.

• Error bars should be included for lag-time and other extracted parameters.

We have included the standard deviations in the tables (see tables S5, S6, S7). We have highlighted the statistically significant changes in the figures. We have plotted all figures per the standard convention, which is to show the raw data points with the mean so the reader can see the scatter.

o Including many stats in the graphs would reduce the need for long and busy tables in the main manuscript, which can be included in the supplementary file.

We have moved all the tables to the supplement as requested by several of the reviewers. We have depicted any statistically significant changes in the figures.

• The authors include summarized growth metrics in Figure 1. Please carry these throughout the manuscript in addition to lag time – it would greatly aid comparison between strains and conditions.

We have only shown lag time in Figures 3-5 because all other growth metrics did not change. The figure was getting too crowded if we included all the metrics. All the metrics are provided as tables in the supplement (see tables S5, S6 and S7) as well as described throughout the results.

- The methods refer to a "custom Matlab script" for growth curve fitting. Please make this available via GitHub or other code-sharing mechanisms. It is difficult to assess the fits to the data otherwise.

We have provided the link to the Github page (see line 403 in the Methods).

- In many figures, the double mutants are presented separately from the single mutants, which, if combined, would allow for better comparisons and improve clarity and readability, since the same WT curve is used in both panels.

We also have the WT strain as an internal reference in all growth curves so that growth can be compared to that strain. If we plot the single and double mutants together then, even with a single replicate, the plots are too crowded to distinguish between the strains. We prefer to keep the single and double mutants separate as shown and none of the other reviewers raised this issue either.

- The titling of panels should be consistent. Some figures (3) have titles where the stressors are labelled, and other figures do not have panel titles. Including a brief title would increase figure readability.

We have provided titles wherever needed and clarified a few details in the figure legends to address this reviewer's comments.

- Why were some experiments conducted in Balch tubes and others in a microplate reader? What is the difference in growth under the same conditions between these assays? It's hard to compare the growth of mutants in different conditions when there's also different assay types.

We have described this detail in the methods (see lines 362-426). Growth on microplate readers cannot be conducted with volatile substrates (like methanol) or substrates that have a doubling time >12 hours due to evaporation (like acetate). Hence, growth on acetate and methanol were conducted in Balch tubes. We did not compare growth in microplate readers to growth in balch tubes in our analysis. All experiments shown in Figures 1, 3, 4, 5 were conducted in a microplate reader and are comparable. Again, we have clarified these experimental details in the methods section (see lines 362-426).

Cell phenotype characterization

- OD measurements alone do not distinguish between viable cells, dead cells, or cells with morphological defects; CFU/mL counts would therefore provide an important validation of the reported growth phenotypes.

As much as would have liked to do so, ee cannot provide CFU/mL counts unfortunately as the plating efficiency of *Methanosarcina* is known to not be reliable enough for CFU measurements (the error is ~3-4 orders of magnitude, if not higher, see Figure 2A in this manuscript https://www.pnas.org/doi/epdf/10.1073/pnas.1618596114). The only reliable viability assay for methanogens is to use F420 fluorescence as we have reported in Figure S9.

o The authors nicely demonstrated cell viability using F420 fluorescence and cell shape visualization under the stationary phase. Extending these viability and cell shape assessments to the other growth conditions and to the DNA-binding domain mutants would further strengthen the conclusions, because OD-based growth curves cannot exclude contributions from non-viable cells or altered cell morphology that may inflate or confound optimal density measurements.

We thank the reviewer for their insightful comments. We did not detect any obvious trends on the other stress conditions that would necessitate a follow-up cell viability assay. These assays are non-trivial to perform and were only conducted under extended lag phase conditions where we observed a clear trend of shorter lag for several mutants. We have also validated the assay by comparing cells treated with a translation inhibitor (puromycin) with untreated cells (see Figure S9). We hope to use this assay in future work for other mutants, but we are not clear on how it can be applied further in this study.

- Width measurements alone are not standard. Morphology should include aspect ratio, area, o Since M. acetivorans has circular cells, this concern could be ameliorated if the authors mentioned this as a rationale for using width as a metric, but suggest that this process of counting width may be automated to reduce user error.

Thank you. We have modified our methods to clarify that we measured width assuming that *M. acetivorans* has circular cells (see lines 447-448 in the methods).

o Increasing cell count (~300) per analysis is necessary to strengthen statistical power.

We imaged at least three random fields and at least 250 cells per condition (see lines 470-471) in the methods.

Experimental rationale and support

- The choice of specific stress conditions (e.g., UV, high temperature, mitomycin C, 21 days stationary phase) should be explained and ideally linked to ecological or physiological relevance for M. acetivorans.

Thank you for pointing this out. We have added more details re: the rationale for picking the stress conditions in the results (see lines 170-177) to address the comments.

- Some early growth experiments appear to use different media without a rationale or mention in the text. Can the authors explain?

See our response to the comment on Balch tubes and microplate readers above. There is a clear rationale for when growth was conducted in Balch tubes (methanol and acetate medium) and microplate readers (all other conditions). We have clarified this in the methods section (see lines 362- 426) and have not made any comparisons across different growth conditions.

- Inclusion of predicted overlaid structures between Mc1A and Mc1B, as well as DNA-binding interface predictions and models, would provide mechanistic grounding to the hypotheses and could motivate which biochemical assays would be most informative.

We have provided more information re: DNA binding by MC1 proteins in the introduction and have highlighted the residues required for this process in Figure S1.

- The RNA seq experiment was performed on "optimal growth conditions", where there were no noted differences in the growth of the WT vs mutant strains, also without any functional categories enriched. If time or future studies allow, could the authors perform RNA-seq in the stress conditions where the greatest growth phenotypes were shown? Inclusion of the double mutant strains in RNA-seq experiments would be informative.

This is an interesting idea. Since the double mutants did not have any growth defects under optimal conditions and RNA-seq of the single mutants did not provide any clear patterns of gene expression, we decided against investing more money in RNA-seq (not a cheap experiment, especially when conducted in triplicate per condition per mutant) for the double mutant or for the single mutants under stress conditions.

- This could strengthen the rationale and conclusions that these proteins might regulate the expression of stress-related genes or global pathways. If not, could they instead discuss this in the text?

Please see our comment above. The only condition under which we detected a phenotype for the histone and NAP mutants was during extended stationary phase where no new RNA is being transcribed. Hence we did not think that RNA-seq would be a valid assay to understand how cells survive in stationary phase.

Data presentation, accuracy, and transparency issues

- Multiple figure titles are mislabeled, and figure citations in the text do not match the correct panels (e.g., Figure 3C, line 144, cited as UV experiment when UV experiment is 3E/F). This occurs repeatedly (line 139 (needs also panel B), line 140, 142). Also, when referencing a subsection of a figure (e.g., only Figure 4D), this should be clearly indicated (line 165).
- Figure S7A has a mismatched label and legend title and lacks clarity in how it relates to mutant graphs, including how it was created.
- These issues need to be addressed to provide a fully confident assessment of the data.

We apologize for this and have rectified all these typos and several others throughout the text.

This study addresses an exciting question and could provide meaningful insight into archaeal chromatin biology. However, the current form contains issues in data visualization, accuracy, and interpretation that must be resolved before the conclusions can be evaluated reliably.

Reviewer #2 comments

Reviewer #2 (Comments for the Author):

General Comments:

In archaeal species that express an abundance of archaeal histones in vivo, the resultant histone-based archaeal chromatin architectures underly one of the key attributes shared between many archaea and all eukaryotes. As such, the archaeal histone-based chromatin systems that most closely mimic eukaryotic histone-based chromatin perhaps dominant the current published landscape. That said, important and significant differences have already been noted in the literature regarding the essentiality and impact of different histones and NAPs in diverse archaeal lineages, particularly in respect to relative in vivo protein expression levels of histones and NAPs (a topic unfortunately missing in the current manuscript). Baskevitch and Nayak present a primarily genetic and phenotypic investigation into three nucleoid-associated proteins (NAPs) in the model species M. acetivorans. This work has been presented in more preliminary forms at major meetings in the field and has generally been positively received. The major findings (each NAP is non-essential, one Mc1 protein must be retained for viability, and mild to perhaps unusual phenotypes resultant from deletion) largely support the previously established contradiction of archaeal NAPs and histones, namely that in some species - particularly thermophilic and hyperthermophilic species - NAPs and histones are often essential, are expressed at quite high levels, and seemingly act across the entire genome, whereas in other species wherein NAPs and histones are lowly expressed (or when histones are not even encoded), the disruption of NAPs or histone expression results in phenotypes and consequences more commonly assigned to site-specific DNA binding proteins. The manuscript, in general, currently lacks reference to many of the foundational studies of archaeal encoded NAPs and their interplay with archaeal histones, as well as references to more recent reviews of archaeal histones, genomic architectures, and the impacts of chromosomal architectures on gene expression. I thus foundationally disagree with the repetitive statements suggesting that the combined architectural roles of NAPs and histones in the same species have not been studied or are ill-defined. The results appear to support a rather long-running theme regarding the disparate roles of NAPs and histone proteins in regulating archaeal gene expression and genomic architecture, based at least in part, on the in vivo protein levels of such. This critique is not a fatal flaw of the manuscript, but rather a suggestion to ensure that existing literature and theories from such are more properly captured in any revised version of this text and that the concept of alternative roles of archaeal histones and NAPs is not unique to this manuscript.

Investigations into nucleoid structure of methanogens is perhaps understudied compared to other archaeal lineages, although at least two very closely related members of the Methanosarcina have been previously experimentally interrogated with respect to histone-dependence and phenotypes resultant from deletion of such (M. mazei) and NAP- and histone-protein abundance (M. barkeri). Note that histone and NAP abundance in the closely related M. barkeri is exceptionally low, implying that NAPs (including Mc1 and HHmA) may not be predicted to have significant impacts on viability or phenotype. The novelty of the current manuscript, in large part, thus stems from the co-evaluation of phenotypes resultant from deletion of two Mc1 isoforms, with Mc1 proteins being widely conserved NAPs (at the genome annotation level) in the Methanotecta and MG-II archaea. The manuscript would thus be greatly improved by a greater

emphasis on the putative importance of Mc1 proteins, as only a single sentence in the introduction (lines 59-61) even references prior work on the DNA binding parameters of Mc1. The average reader is not sufficiently informed regarding Mc1A and Mc1B to establish if these NAPs are the most critical NAPs for M. acetivorans nor pointed to established literature that demonstrates that mc1 genes are minimally-well conserved across major archaeal lineages. Even as someone who has contributed to the field of archaeal histone and NAP literature, I was rather lost regarding the evidence (beyond conservation at the genome level) of why Mc1A/Mc1B were targeted and what evidence exists within M. acetivorans for a major role of Mc1A or Mc1B, alone or together, in establishing chromatin architecture(s) within M. acetivorans. Review of references 18-22 speak to histone-based work in M. mazei (ref 18) and purified biochemical and biophysical investigations of Mc1-DNA binding (refs 19-22), but there are no references to the physiological roles of Mc1. In this respect, the current manuscript aims to fill this knowledge gap, but without reference to why Mc1A/Mc1B should be investigated, the average reader is unlikely to understand the importance of the current investigations. Repeated claims that Mc1 may compact the genome should be removed, as no evidence for such exists; Mc1-DNA binding is known to bend DNA sharply, but the impacts of Mc1 on compaction, in vitro or in vivo, are not demonstrated here or referenced in the established literature.

We thank the reviewer for their comments. A major concern raised by this reviewer is about the introduction and the background provided. We have addressed this concern by completely rewriting this section. We have now discussed a lot more of the background information requested by this reviewer re: MC1 proteins.

Major comments:

1. The authors are genetic experts, and the strain constructions are largely well done. According to Table S1, in addition to the desired clean deletion(s) of the targeted loci for the individual deletion strains, in two of the three individual deletion strains, some regions of the genome appear (although it is not entirely clear to me) to have insufficient sequence coverage to ensure that additional mutations were not observed. In strains deleted for the histone and one Mc1 isoform, at minimum, a second-site mutation appears to be present (a single bp deletion in adenine deaminase which likely results in a frameshift and thus is likely to be completely inactivating). These potential/known undesired genomic modifications in multiply-deleted strains are carefully excluded to not technically be at odds with the text in line 89, but are hardly forthcoming. These mutations must be discussed, as they may be relevant to the phenotypes described. How can the authors assure that the phenotypes observed are not due to these second site mutations? What level of genomic coverage was used to establish the absence of other modifications to the genomes compared to the genome of WWM60?

We thank the reviewer for their comment. Short reads (~100-200 bp) obtained by Illumina sequencing are hard to align to repeat regions in the genome and some of the common computational tools will show certain repeat regions as regions of missing coverage because they randomly align these regions to different parts of the genome. This is an artefact of sequence alignment rather than a biological detail. We did not want to cherry pick the output of the computational program but have now removed any artefacts generated by it. We know these are artefacts because we can detect these missing regions by PCR.

In previous work, we showed that null mutations in *ade* routinely occur when *M. acetivorans* is grown in media supplemented with 8-ADP (8-aza-2,6-diaminonpurine sulfate) to cure the CRISPR-editing plasmid (see reference # 32 in the text). The *ade* mutations have no detectable impact on growth under laboratory

conditions and can be ignored for the purpose of this work. We have also added this to the results section for clarify (see lines 101-106 in the text).

2. A rather large aspect of essentially all the phenotypic evaluations of the deletion strains is investigated is a delayed restart of measurable growth following extended nutrient limitation or stress. The authors do measure cell shape and size, and the results are important in validating their conclusions. The phenotypes described in text lines ~137-145 are mixed in nature and report observations without causality. These discrepancies and differences in growth rates in different media and following different stresses go without further discussion and this limits enthusiasm regarding the value of the observations themselves. What is learned or inferred from these mixed phenotypes? The revised text should attempt to rationalize the mixed phenotypic behaviors observed, as detailing these phenotypes without explanation will leave the naïve reviewer unclear of the importance of such observations.

We would argue that there are some differences in the stress response data but nothing meaningful that was worth dwelling on beyond what is stated in the text. At this point, we cannot say anything about why these phenotypes manifest the way that they do. But, the lack of a rational explanation does not discount the validity of these data itself. We hope that these data will provide some value to future work on these proteins.

3. A parsimonious explanation for the change of timing from the exit of stationary phase (the primary descriptor of phenotype in this work) is a strain-dependent difference in the number of viable cells in extended stationary phase. More or less viable cells used for re-inoculations would seemingly result in exactly the observed differences in lag before growth restart. Instead of directly measuring colony forming units (CFUs) of cultures prior to inoculation into nutrient-containing media (which has been the experimental norm for decades), a rather obscure (in my opinion) metric of F420 fluorescence is used as a proxy for cell viability. Intact cells are the readout of this metric which is unfortunately equated to viable cells, and this may unfortunately not be the case. To orthogonally-validate the key results of the phenotypic analyses, it appears essential to establish viable cell counts for each strain following extended incubation in nutrient limiting conditions. I cannot advocate for publication of this work without these critical evaluations.

We disagree with the reviewer's assessment here. F420 is not an obscure metric and is repeatedly used to measure the viability of methanogens. Please see these references:

1. Lambrecht J, Cichocki N, Hübschmann T, Koch C, Harms H, Müller S. 2017. Flow cytometric quantification, sorting and sequencing of methanogenic archaea based on F420 autofluorescence. Microbial Cell Factories 16:180.
2. Khanum S et al. 2026. Detection and quantification of rumen methanogens using F420 autoflourescence profiling with spectral flow cytometry. Appl and Environ Microbiol e01416-25.

As noted in our response to reviewer #1 above, we cannot use CFUs as the plating efficiency of *Methanosarcina* is quite low and variable even between technical replicates so F420 is really the only tool available to use. Some researchers have used live/dead stains in the past but we would argue about the validity of these methods because these stains primarily use membrane integrity as a metric for viability. F420 is a far better metric in that regard. In addition, any stains that use a pH gradient to measure viability do not work for these methanogens as they maintain a Na+ gradient. We also looked into a luciferase-based

assay to measure ATP levels in vivo but were discouraged by extremely noisy measurements across technical replicates. The reviewer's point is well taken but there is no other readout better than F420, which is far from obscure, as they opine.

4. A significant concern seemingly arises regarding the transcriptomic evaluations. This concern is twofold. First, the manuscript (lines 115 and again on line 396) reports that log2 fold changes of -/+ 0.1 were considered significant. The log2 of 0.1 equates to -3.32, which makes little sense in any scenario. The norm of many manuscripts is to set a threshold of log2 changes of 1.0 or greater. Given that I cannot understand a log2 threshold change of 0.1, I assumed that the authors meant log2 of 1.0. Secondly, with this in mind, the noted undesired frameshift in adenine deaminase in several strains might have been expected to manifest itself in the transcriptome analyses. If a frameshift is not sufficient to drive significant gene expression changes, how does one then interpret the seemingly insignificant changes to the rest of the transcriptome?

The reviewer seems to have a misunderstanding here and we have now clarified this in the text (see below and lines 126 and 132). The frameshift mutation in adenine deaminase is in a double deletion mutant which was not used for RNA-seq. All the RNA-seq was conducted with the single mutants that do not have frameshift mutations in adeC.

'Next, we identified genes that were differentially expressed in the DBP deletion strains relative to the WT. We designated a gene as being differentially expressed (DE) if it had a log_2 fold change > +0.1 or < -0.1, i.e. ~7% change in expression, and was statistically significant (q-value < 0.05). We chose a log_2 fold change of 0.1 because < 10 genes were differentially expressed in each strain using the typical log2 fold change cutoff > + 1 or < - 1. We reasoned that small changes in the expression of genes involved in DNA repair or regulation might have a meaningful impact on physiology and thus could be relevant for our work.'

5. While efforts to establish changes in the transcriptome are generally meritorious, it is rather well established that changes to the transcriptome and proteome are poorly correlated in many archaeal species. NAPs and histones, at least in vitro, are hardy proteins that resist degradation and thus may be anticipated to be long-lived in vivo. Given the pioneering work from Warnecke and Hocher that details the importance of NAP and histone abundance at the protein level in diverse archaeal lineages, it is perhaps worth investigation in M. acetivorans. If Mc1a and Mc1b are not abundant proteins (as would appear to be the case based on studies in M. barkeri), deletion of such would not be anticipated to drive much of a phenotype. Based on measurements of histone abundance in M. barkeri, the same minimal impact may have been anticipated for M. acetivorans . In this regard, I again return to my general query regarding the known importance of investigating Mc1 proteins in M. acetivorans. The comments in the discussion, lines 195+, refer to perceived abundances based on RNA levels which unfortunately cannot be demonstrated to accurately reflect protein levels in vivo.

We have rewritten the discussion to soften any functional claims about the MC1 proteins. We hope this addresses the reviewer's concerns.

6. Regarding protein expression levels, the efforts near the end of the results to complement strains by

expression of the individual deleted genomic loci do not take into account the level of ectopic expression compared to genomic expression of any of the DBPs. It is known that overexpression of archaeal histones can be phenotypic on its own, and that expression of mutants that lack critical DNA binding residues reduces these observed phenotypes. Thus, although the authors provide evidence that the DBPs (with epitope tags) are expressed, they do not establish if the ectopic-expression restores, in part, in total, or even radically overexpresses the DBPs at different stages of the growth cycle. Here it appears that additional studies will also elevate the manuscript. It is also well-established that the addition of epitope or affinity tags to archaeal histones can dramatically impact DNA binding capacities of archaeal histones. Do the authors have any evidence to support that the epitope tag additions to their DBPs are innocuous? Prior work in Methanogens from the Reeve lab would suggest otherwise.

We have shown that complementing deletion mutants with an epitope tagged version of the gene rescues the lag phenotype, which indicates that the gene is functional (see Figure S11). We have also shown that the addition of the tag does not impact the stability of the protein, in terms of its net yield after expression from the same inducible promoter (see Figure S10) for the WT and mutant alleles. We hope these controls satisfy the reviewer.

7. Supplemental figure 14 b) and d) illustrate growth curves post-28-day stationary phase incubation for DBP mutants that lack critical DNA binding residues, specifically Mc1b and HMaA. I would recommend further discussion on this mutated HMaA strain. Authors state that the phenotype of strains expressing these mutant DBPs phenocopy the deletion mutants. This is true of MC1b mutants; however, HMaA mutants have a different phenotype than both the HMaA deletion strain and control strain (as shown in Table 4, it is a statistically significant difference). This phenotype is not discussed further.

We don't have much to discuss beyond fact that the mutant allele may still retain the ability to bind DNA weakly. We have mentioned this in the discussion (see lines 235-238).

8. In lines 180+ the differences with M. mazei are discussed. While presented as radically different, I disagree. In both methanogens that single histone-encoding locus is dispensable and the resulting deletion strains are viable and mildly-phenotypic. It is true that the technologies available for strain construction have radically shifted in the last two decades.

We disagree with the reviewer's assessment. The *M. mazei* paper is titled 'Deletion of the archaeal histone in Methanosarcina mazei Gö1 results in reduced growth and genomic transcription' and the major claim of this work is that the disruption of the histone gene leads to severe growth defects on TMA and methanol, which we did not observe. In addition, they observe severe sensitivity to UV shock, which we did not observe either. We stand by our assessment here.

9. In the final paragraph of the discussion, histones have always, in my opinion, been considered NAPs with a specialized fold. Histone proteins are defined by the histone-fold, and thus are NAPs. This is reminiscent of the adage that "all squares are rectangles, but not all rectangles are squares". There seems to be little value in attempting to remove the archaeal histone designation given that the moniker refers to the fold, not the function of the protein. It is again important to alter the text such that the current results fall in line with prior designations that histones and NAPs in diverse archaea play diverse roles, rather than attempting to assert that this manuscript is the first to point out this phenomenon.

We have rewritten the discussion to soften many claims and address the reviewer's concerns.

10. As is true for many manuscripts, the current text appears to be a thesis-turned-manuscript and contains a perhaps overabundance of supplementary figures. Figure S1 is an excellent review of Mc1 and HMaA but is essentially not discussed to a level of significant value in the current text. The important data within Figs S2/S3/S5/S9/S11/S14 is seemingly already captured in main text figures. Figure S10 can easily be explained in the M&Ms without a figure. The value of the logo plots in S8 is unknown without some descriptor of sequence-specific binding information of the Mc1 paralogs. Figure S12 should be replaced with a viable cell or colony-forming unit cell count. Figure S13 would be dramatically elevated by generating strains wherein the genomic loci of each DBP was tagged to reveal the relative abundance, rather than simple definition, of ectopic expression. This is unnecessary for publication, but all results regarding complementation are complicated by the unknown level of protein restoration or overexpression in plasmid-containing strains. While well done, the value of S15 to this manuscript is unknown, as a similar description in present in work from Hocher and Warnecke. A similar points is raised for supplemental tables, with ST2 being redundant, and a reference remaining in ST12.

We have removed many supplementary figures or merged them per the reviewer's suggestion.

Minor comments:

1. Within Table S1, the table description appears to include a note mad by authors for themselves regarding "cite BreSeq in the methods" that should likely be removed.

We have fixed this typo. Thank you for pointing this out.

2. Discussion, line 221, the term "art" is seemingly misplaced. Perhaps this is meant to be "work"?

Art is grammatically correct but we have changed the discussion and rewritten this section.

3. Line 296, the composition of CO_2 should be clarified as 20% so as to not confuse with the potential for 20 ppm (as the unit listed for H_2S).

We have fixed this typo. Thank you for pointing this out.

4. Throughout the M&Ms, values and units should be separated (50mM to 50 mM).

We have fixed this typo. Thank you for pointing this out.

5. When John Reeve and colleagues first starting descriptions of archaeal histones they established a naming feature of capital H for histone, followed by the genus/species two letter code of the host organism. In species with multiple histone isoforms, each isoform was designated a letter (A, B, C). Thus, throughout the manuscript, the single histone (H) isoform (A) from M. acetivorans (Ma), should be referred to as HMaA. The two histones from T. kodakarensis are thus HTkA and HTkB. Please correct throughout.

Interesting but we can't seem to find a written record of this convention. We have made the change as requested, though.

6. Within the introduction, line 50, reference 5 is now 23 years out to date and numerous more relevant reviews should be included.

We have rewritten the introduction and added more (recent) references.

7. Within the introduction, line 55, archaeal histones do NOT form nucleosomes. The term nucleosome is most typically restricted for description of the discrete, octameric histone-DNA complex (so far) exclusive to eukaryotes. Archaeal histones can form structures that wrap DNA into a conformation that matches the structure of DNA within the eukaryotic nucleosome, but this is quite distinct from stating that "archaeal histones have been demonstrated to form nucleosomes".

We have rewritten the introduction to address this comment.

Given the text improvements that are readily available, and some valuable orthogonally-validating experiments that will greatly improve the overall conclusions, I recommend that the authors return with a modified manuscript inclusive of additional experiments prior to recommendation for publication.

As has been my norm for ~6 years, I firmly believe in identifying myself in my reviews. I find that in doing so the authors can more appropriately respond to suggestions and critiques, and further, that any noted critiques are more carefully composed during my review. I realize that many journals do not permit or desire reviewers to identify themselves, but I do so regardless. I thank the authors for the opportunity to assist with these studies. I in fact apologize for my extended review, but it is only my goal to improve the manuscript and assist. My best - Tom Santangelo

Reviewer #3 comments

Reviewer #3 (Comments for the Author):

The manuscript JB00559-25 describes a genetic analysis of 2 NAPs and 1 histone protein in M. acetivorans, followed by RNA-seq analysis and phenotypic growth analysis. Key results are the lack of essentiality (with the exception of the requirement of minimally one MC1 copy), minimal effects of gene deletion on the transcriptome and growth phenotype in standard conditions, but phenotypic effects in stress conditions (UV shock) and stationary phase revival. It is an interesting study that is backed up by a large amount of high-quality data, obtained by employing solid research methods. It contributes to the expanding knowledge base of the divergence of non- or less-sequence specific DNA-binding (histone and non-histone) proteins that combine chromatin organization with global gene regulation in archaea. In hindsight, it would have been a better experimental design choice if the authors had selected the cultivation conditions that result in a different growth phenotype for the transcriptomic analysis (UV stress or recovery after stationary phase), so that we might have gotten more insights into the underlying transcriptomic programs underlying the growth advantage phenotypes observed for some of the knockouts. In addition, analysis of DNA-binding properties (in vitro or in vivo with ChIP) could be helpful to further unravel mechanistics of the functioning of the 3 DBPs. But this does not necessarily needs to be addressed within the scope of the current manuscript. I only have some remarks to further improve the text.

We thank the reviewer for their positive comments.

Major remarks

1. The authors are invited to consider moving some of the supplementary figures into the main manuscript, especially Fig. S1, Fig. S6 and Fig. S14.

Since reviewer 2 seems to think there is a lot of redundancy between main text figures and the supplement, we have chosen to keep the main text figures as is. We have made quite a few improvements to the figures and the text that we hope addresses some of the reviewer's concerns.

2. Lines 59-62: The authors are invited to slightly extend their Introduction and to provide more information on which NAPs and histones have been identified in Methanosarcina spp. and what is already known about them. For example, the authors state: "In Methanosarcina spp., histones are also not essential and have been proposed to play an auxiliary role during DNA damage repair. In cases where histones are absent or non-essential, archaeal NAPs, such as Cren7 and Alba, have been implicated in genome compaction instead." (lines 59-62). Does this mean that Cren7 and Alba are present in M. acetivorans/Methanosarcina? How many histones are encoded in the M. acetivorans genome? Is HMm known to play a role in DNA damage repair and, if so, what exactly is that role? Could the authors also clarify what is known about the two MC1 paralogs (structure and function)?

We thank the reviewer for their comment. We have rewritten the introduction entirely to address their comments and concerns.

3. Lines 72-76: In the last paragraph of the Introduction, the authors already disclose the key findings of the study. I would suggest limiting the final paragraph of the Introduction to a clear statement of the research objectives, approach and possibly, the working hypothesis (based on the previous knowledge on MC1s and HMm: do you hypothesize that these DBPs are dispensable and/or have redundant/overlapping functions? Do you hypothesize that their function is linked to stress response and if so, based on which previous findings?) As a reader, I find it preferable if the outcome of the study remains open when reading the Introduction.

Please see our response to the comment above. We have rewritten the introduction and haven't discussed the results to the degree we did before, as requested by the reviewer.

4. Lines 114-116: The authors use a $\log_2$ fold change threshold of 0.1 to designate genes as differentially expressed. This corresponds to an approximately 7% change in expression, which is -to my knowledge- much lower than what is typically used in differential gene expression studies. It is unclear whether such small transcriptional changes are likely to translate into biologically meaningful effects. Could the authors justify this choice of threshold and, ideally, support it with additional rationale (for example, effect size distributions, sensitivity analyses or validation of selected targets)?

We thank the reviewer for their comment. have provided a rationale for this choice in the text (see lines 127-132) and also pasted below. We hope this addresses the reviewer's concerns.

We designated a gene as being differentially expressed (DE) if it had a $\log_2$ fold change > +0.1 or < -0.1, i.e. ~7% change in expression, and was statistically significant (q-value < 0.05). We chose a $\log_2$ fold change of 0.1 because < 10 genes were differentially expressed in each strain using the typical log2 fold change cutoff > + 1 or < - 1. We reasoned that small changes in the expression of genes involved in DNA repair or regulation might have a meaningful impact on physiology and thus could be relevant for our work.

5. Lines 119-120: The authors state: "A response regulator (MA_RS14075) was the only protein-coding gene differentially expressed in both the Δmc1a and Δmc1b mutants (Table S3)." The authors could also refer to Fig. 2B, where this gene is highlighted. In addition, Fig. 2B appears to indicate two other genes that are differentially expressed across the various knockout strains (MA_RS26970 and MA_RS27685). Could the authors comment on these observations and clarify how these genes relate to the statement above?

We have revised our analysis of the transcriptomic data based on the reviewer's comments and would like to apologize for this incorrect statement. Please see the revised statement in the text (163-167) and pasted below. We hope this addresses the reviewer's comment.

Two genes encoding putative smORFs with unknown functions (MA_RS26970 and MA_RS27685) and a partial CDS for a response regulator (MA_RS14075) were differentially expressed in both the Δmc1a and Δmc1b mutant (Fig. 2B). Thus, even though our genetic analyses suggest that the two mc1 genes have some functional overlap, it is not necessarily evident in the global transcriptome.

6. Lines 103-104: The authors state: "To test if the dispensability of individual DBPs stems from functional redundancy, we obtained the global transcriptomic profile of the three single knockout mutants and WT..." (lines 103-104). It is not entirely clear what is meant by "functional redundancy" here and how the authors link this specifically to transcriptome profiling. Later, the authors conclude that "even though our genetic analyses suggest that the two mc1 genes have some functional overlap (Table S2), it is not evident in the global transcriptome" (lines 120-122). Could the authors clarify the rationale here and comment on whether an absence of transcriptome-level changes necessarily argues against functional overlap or redundancy?

We thought we could look for genes that these two proteins might regulate and that shared function might be essential, but that does not seem to be the case since the transcriptomic profile of the deletion mutants has a very small overlap and is quite distinct. We have explained this in more detail in the text (see lines 165-169).

7. Line 125: It is stated: "The DE genes were not enriched for any specific functional category (Fig. S7 and Tables S4-S8)." I would suggest either extending the analysis or toning down this statement, as the current phrasing implies that a formal statistical enrichment analysis was performed, which does not appear to be the case. In Fig. S7, the authors show the distribution of DE genes across COG categories and note that it "largely mirrors" the overall genomic distribution. However, a visual similarity between distributions does not constitute an enrichment analysis unless it is supported by statistics (for example, p values). In addition, it is unclear which gene set was used for Fig. S7. The number of significantly DE genes shown for each knockout strain appears lower than the numbers implied by the criteria described in lines 117-119. Could the authors clarify this discrepancy?

We have removed this analysis as we deemed it was too preliminary based on the reviewer's comments.

8. Lines 126-128 and Fig. S8: The authors state: "A TTTTC motif was enriched in the promoter region of DE genes for the Δmc1b mutant, which is consistent with previous findings that this protein preferentially binds certain DNA sequences (Fig. S8)." Could the authors specify which criteria (for example, E-value or other significance threshold) were used to consider the TTTTC motif significant, and why none of the other candidate motifs were retained? In addition, the authors are invited to examine the TTTTC motif results in more depth. Does the inferred DNA-binding preference indeed match the previously reported binding specificity cited here? Are the 676 detected sites evenly distributed across the promoter regions of all DE genes, or are they driven by a subset of promoters with multiple motif occurrences? If multiple occurrences are present in a promoter region, do they show any regular spacing? Finally, is motif enrichment higher close to the start codon and core promoter elements compared to more distant upstream regions (given that archaeal transcription regulators typically bind near promoter elements) and is a 6-bp motif plausible given the expected protein-DNA interface and binding site size (cfr. protein structure presented in Fig. S1)?

We have removed this analysis as we deemed it was too preliminary based on the reviewer's comments.

9. Lines 133-134: The authors state that "the histone deletion mutant of M. mazei Gö1 was shown to be more sensitive to UV shock than the parent strain." It is interesting that, in this study, the histone deletion

mutant of M. acetivorans appears to perform better after UV shock than the WT. Could the authors comment on how these observations in the 2 studies can be reconciled? For example, are the histones in M. acetivorans and M. mazei clear orthologs and what is known about their sequence identity, conservation of key DNA-binding residues and likely functional homology? In addition, were the UV shock conditions and experimental set-up comparable between the two studies?

This is an interesting point. We consulted with the authors of the previous work and have performed our experiments the same parameters to the extent possible. However, our histone deletion mutants don't seem to be sensitive to UV shock. We don't yet know why. One plausible reason could be how the mutants were constructed (as described in the text- see lines 255-262) strain has a pac cassette and puromycin in the growth media, while ours is a clean deletion. Alternately, there are real biological differences between the two strains in terms of the physiological role of histones. The two histone genes are <95% identical at the amino acid sequence level.

10. Lines 166-168 and Fig. S1: Could the authors clarify the rationale for the selected mutations? For HmaA, residue R15 does not appear to be conserved in M. fervidus HmfB, so it is unclear why this position was prioritized over other conserved residues. In addition, why was an alanine-scanning approach not consistently applied for the R15T, R21S and T56K substitutions? Does this selection reflect specific prior insight into the protein-DNA interaction interface and the type of interactions (e.g. electrostatic)? Finally, regarding the Mc1b R25A mutation, I might be mistaken, but in the alignment shown in Fig. S1D the conserved R residue appears to be at position 26 rather than 25. Could the authors verify the residue numbering?

We have provided more of a rationale for each set of mutants. See lines 224-231. Rather than alanine scanning we used exactly the same point mutations that were made in previous studies that used EMSas to show that reduced DNA-bindinng affinities. Re: Figure S1, we have verified the residue numbering and fixed the typo.

11. Lines 195-201: Some of the statements in this Discussion are quite bold and could be perceived as overinterpretation. For example, "MC1a ... is likely the primary driver of genome compaction in M. acetivorans." Based on the results presented in this paper, which do not include a direct study of DNA-binding properties or effects on DNA compaction, it is difficult to make this statement as written. Likewise, "it is plausible that MC1b is a master regulator of many different regulons as 16 genes predicted to bind DNA and/or regulate transcription are differentially expressed in the Δmc1b mutant" may be perceived as too strong, since differential expression alone does not necessarily imply direct regulation or "master regulator" activity. I suggest tempering the wording and framing these points more explicitly as hypotheses that are consistent with the observed transcriptomic patterns, unless additional evidence can be provided to support the stronger conclusions.

We have rewritten large sections of the manuscript to temper wording and soften claims.

Minor remarks

- Fig. S2: Unless I'm mistaken, there is no reference of this supplementary figure in the main text.

Yes, we have fixed this typo and added a reference to this figure now (see line 91).

- Legend Fig. S6: typo "Expression of each"

We have fixed this typo.

- Line 116: The sentence "At a global level, each DBP has a unique transcriptional profile" is ambiguous and could be read as referring to the DBP gene's own expression profile, rather than the transcriptome of the deletion strain. Please consider rephrasing, for example: "At a global level, each DBP deletion strain exhibits a unique transcriptional profile."

We have fixed this typo (see line 133).

- Line 210: "Solfolobus" → "Saccharolobus"

We have fixed this typo (see line 276).

- Fig. S1: typo "imporant" → "important"

We have fixed this typo.

Re: Spectrum02785-26R1 (**Histones and nucleoid-associated proteins in *Methanosarcina acetivorans* mediate recovery from stress and stasis**)

Dear Prof. Dipti D Nayak:

Your manuscript has been accepted, and I am forwarding it to the ASM production staff for publication. Your paper will first be checked to make sure all elements meet the technical requirements. ASM staff will contact you if anything needs to be revised before copyediting and production can begin. Otherwise, you will be notified when your proofs are ready to be viewed.

Data Availability: ASM policy requires that data be available to the public upon online posting of the article, so please verify all links to sequence records, if present, and make sure that each number retrieves the full record of the data. If a new accession number is not linked or a link is broken, provide production staff with the correct URL for the record. If the accession numbers for new data are not publicly accessible before the expected online posting of the article, publication may be delayed; please contact ASM production staff immediately with the expected release date.

Publication Fees: For information on publication fees and which article types have charges, please visit our website. We have partnered with Copyright Clearance Center (CCC) to collect author charges. If fees apply to your paper, you will receive a message from no-reply@copyright.com with further instructions. For questions related to paying charges through RightsLink, please contact CCC at ASM_Support@copyright.com or toll free at +1-877-622-5543. CCC makes every attempt to respond to all emails within 24 hours.

ASM Membership: Corresponding authors may join or renew ASM membership to obtain discounts on publication fees. Need to upgrade your membership level? Please contact Customer Service at Service@asmusa.org.

Embargo Policy: A press release may be issued as soon as the manuscript is posted on the Spectrum Latest Articles webpage. The corresponding author will receive an email with the subject line "ASM Journals Author Services Notification" when the article is available online.

Cover Image Submissions: If you would like to submit a potential Cover Image, please email a file and a short legend to spectrum@asmusa.org. Please note that we can only consider images that (i) the authors created or own and (ii) have not been previously published. By submitting, you agree that the image can be used under the same terms as the published article. Image File requirements: TIF/EPS, 7.5 inches wide by 8.25 inches tall (at least 2,250 pixels wide by 2,475 pixels tall), minimum 300 dpi resolution (600 dpi preferred), RGB, and no figure elements, e.g., arrows or panel labels. The legend should be a short description of the image, 1-2 sentences recommended. Please use the ASM Journals Cover Image Sizing Tool (https://journals.asm.org/pb-assets/html/asm-cover-sizing-tool.html) to ensure that your proposed cover image meets our size requirements.

The ASM Journals program strives for constant improvement in our submission and publication process. Please tell us how we can improve your experience by taking this quick Author Survey.

Thank you for submitting your paper to Spectrum.

Sincerely,
Blaire Steven
Editor
Microbiology Spectrum
