# Supplementary material for: Histones and nucleoid-associated proteins in Methanosarcina acetivorans mediate recovery from stress and stasis
Source: Microbiol Spectr. 2026 Oct 14;14(11):e02785-26. doi: 10.1128/spectrum.02785-26 (PMC13729102; doi:10.1128/spectrum.02785-26)
Supplement: Supplementary file 2 [file spectrum.02785-26-s0001.pdf]

Supplementary figures

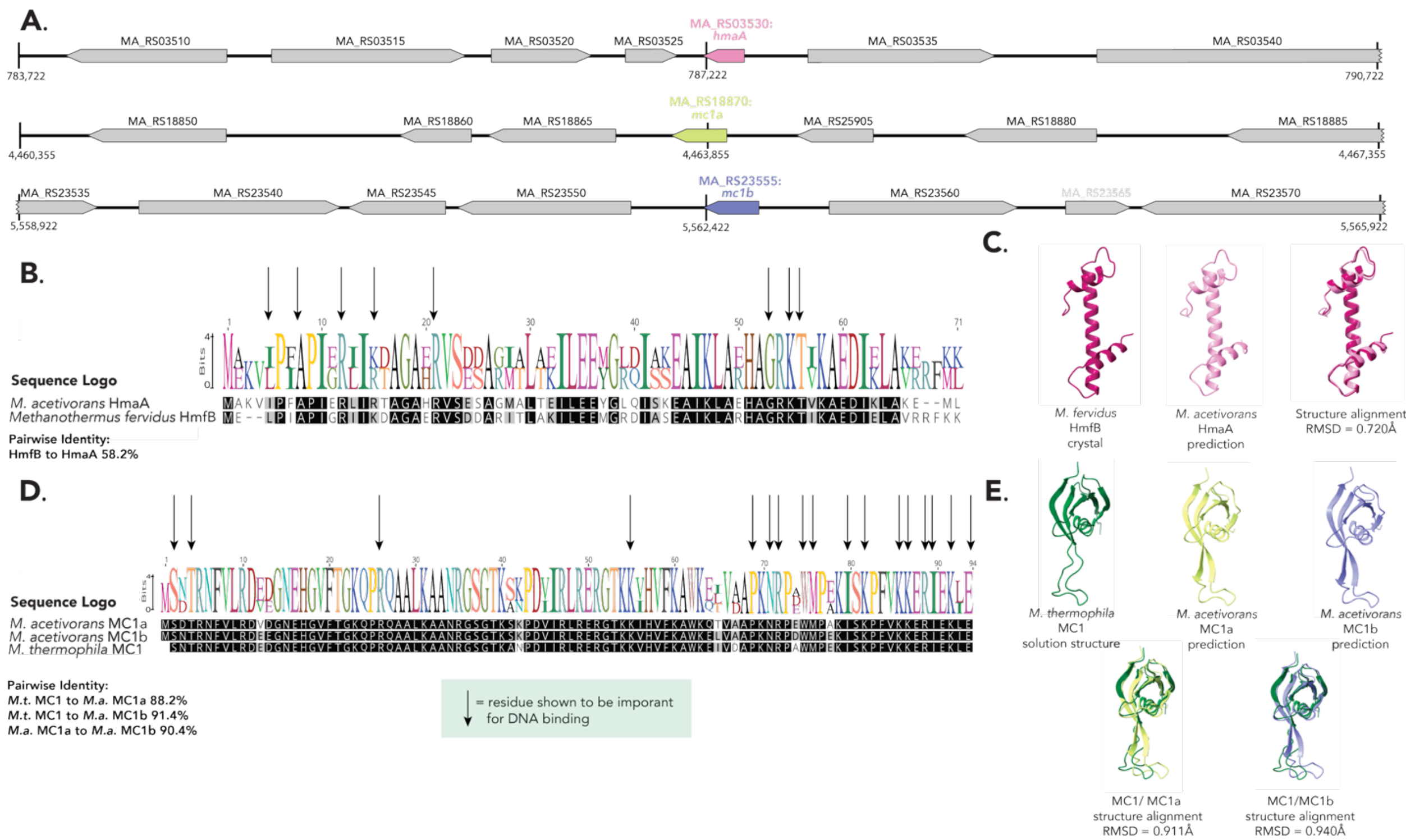

Figure S1: ***M. acetivorans*** **encodes one histone and two MC1 paralogs with conserved structure and functional residues. A**. Schematic showing genomic location of genes encoding known DNA-binding proteins (DBPs) in *M. acetivorans* C2A. A region of 7000 base pairs surrounding each DBP encoding gene is shown with unrelated open reading frames depicted in gray. Genomic coordinates for each region are noted. DBP genes are highlighted in color. Locus tags for all open reading frames are noted. **B.** Amino acid alignment of *M. acetivorans* histone HmaA with the model archaeal histone *Methanothermus fervidus* HmfB. Black arrows indicate residues important for DNA-binding as determined by Pereria & Reeve[13]. Pairwise identity between HmfB and HmaA is noted below the alignment. **C.** Crystal structure of HmfB[14] compared to AlphaFold2 prediction[44] for HmaA. A structure alignment between HmaA and HmfB is shown and Root Mean Square Displacement (RMSD, see Methods) is noted below the alignment. **D.** Amino acid alignment of *M. acetivorans* MC1a and MC1b with *Methanosarcina thermophila* CHTI55 MC1. Black arrows indicate residues important for DNA-binding as shown by Paquet & collagues[28] as well as Loth and colleagues[29]. Pairwise identities between *Methanosarcina thermophila* MC1 and *M. acetivorans* MC1a and MC1b, respectively, are noted below the alignment. In addition, pairwise identity between *M. acetivorans* MC1a and MC1b is noted below the alignment. **E.** Nuclear Magnetic Resonance (NMR) solution structure of *M. thermophila* MC1[28] compared to AlphaFold2 predictions for *M. acetivorans* MC1a and MC1b. Structure alignments between *Methanosarcina thermophila* MC1 and *M. acetivorans* MC1a and MC1b, respectively, are shown with RMSD noted below each alignment (see Methods for details).

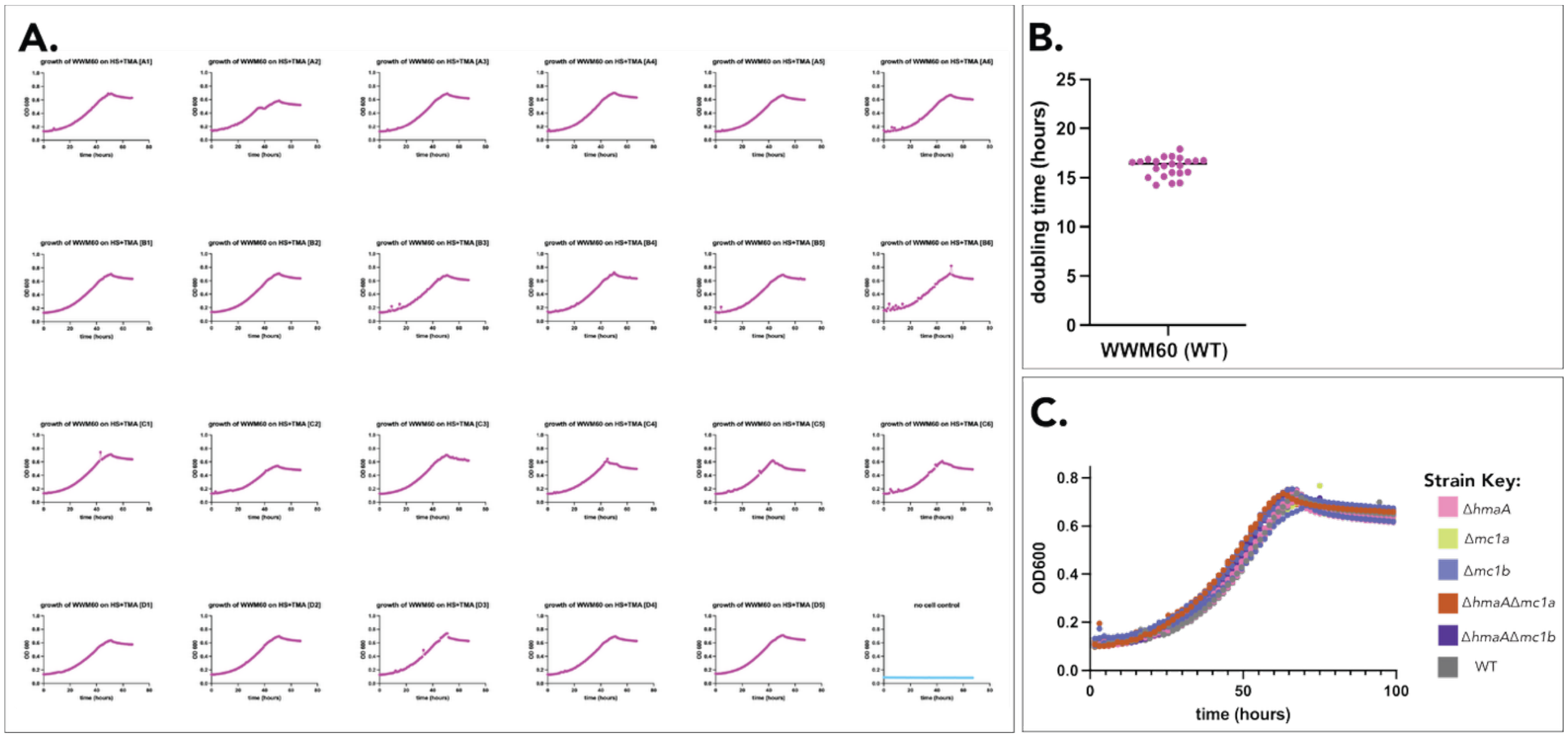

<u>Figure S2:</u> Anaerobic high-throughput growth measurements for *Methanosarcina acetivorans*. A. Growth curves of the parental strain used in this study (WWM60) measured as a change in optical density (at 600 nm) over time in high-salt (HS) minimal medium supplemented with 50 mM trimethylamine (TMA) as the growth substrate. Growth is tracked in individual wells of a 24-well plate sealed with optical film. The plate is incubated in a plate reader inside of an anaerobic chamber (see Methods for more details). Panel **A** shows growth plotted for each individual well in a representative experiment. **B.** Plot showing doubling time as calculated for each individual well in the experiment shown in panel **A**. The horizontal bar represents the mean of 23 replicates. **C.** Growth curves of the DNA-binding protein (DBP) deletion mutants shown in Figure 1. DBP deletion mutants are depicted by colored circles and shown in comparison to the parental strain (WWM60, denoted as wildtype or WT, gray circles). Growth is measured as a change in optical density (at 600 nm) over time in high-salt (HS) minimal medium supplemented with 50 mM TMA as the growth substrate. Growth parameters calculated from these curves are shown in Table 1. Data included in panel **C** here is also depicted in main text figure **1A**, but repeated here for the purposes of comparison.

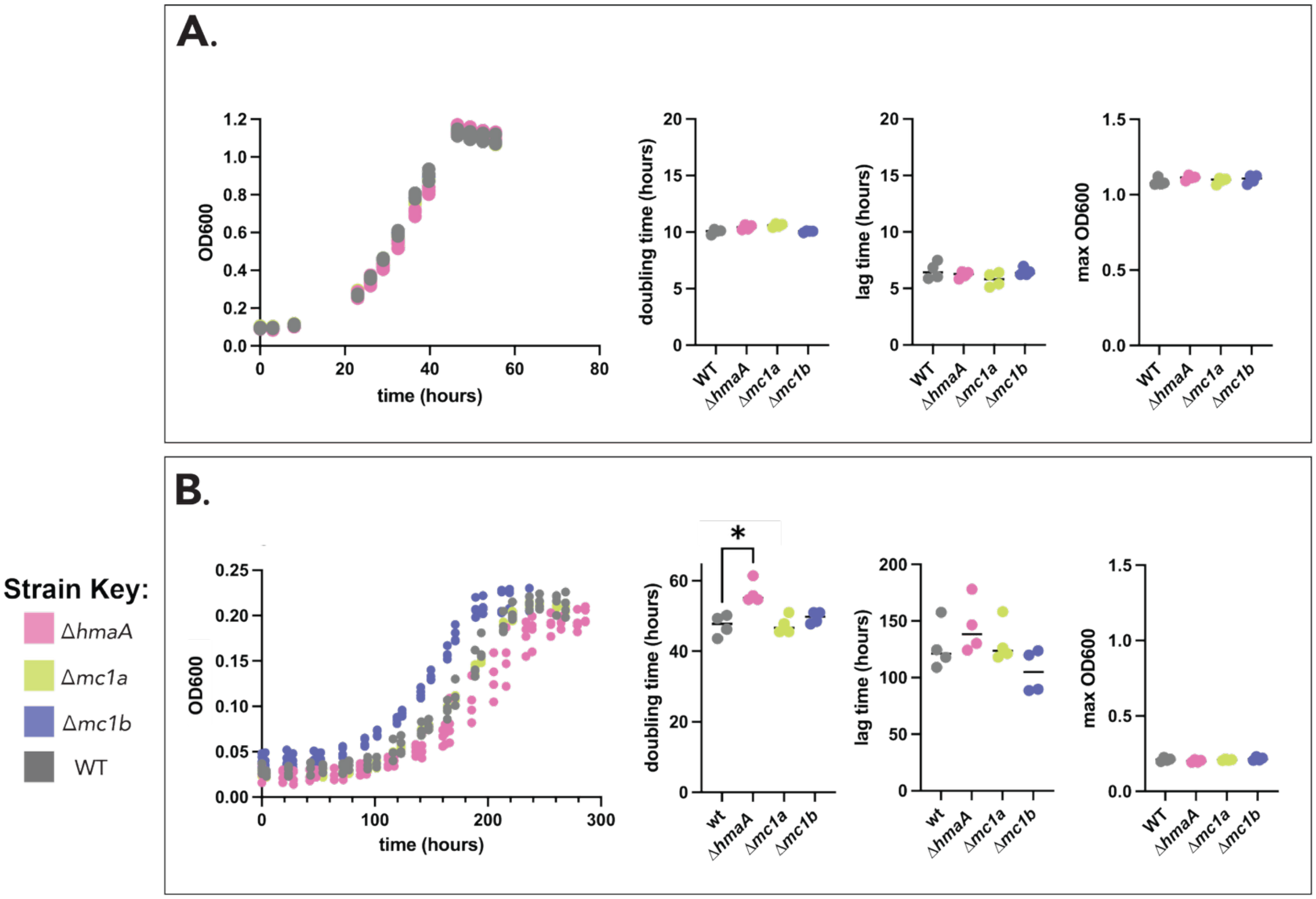

<u>**Figure S3:**</u> **DNA-binding proteins (DBPs) are not individually essential in *M. acetivorans*.** Growth curves represented by changes in optical density (OD600) over time for single DBP deletion mutants (colored circles) and the parent strain (WWM60; denotated as wildtype or WT, gray circles) in high-salt minimal medium with **A.** 125 mM methanol or **B.** 40 mM sodium acetate. Doubling time (in hours), lag time (in hours), and cell yield (represented as max OD600) were calculated from four replicate growth curves per strain per growth condition. The horizontal bar represents the mean of the replicates. Statistically significant differences relative to WWM60 ($p \leq 0.05$) based on a one-way ANOVA with Dunnett's multiple comparisons test are depicted with an asterisk.

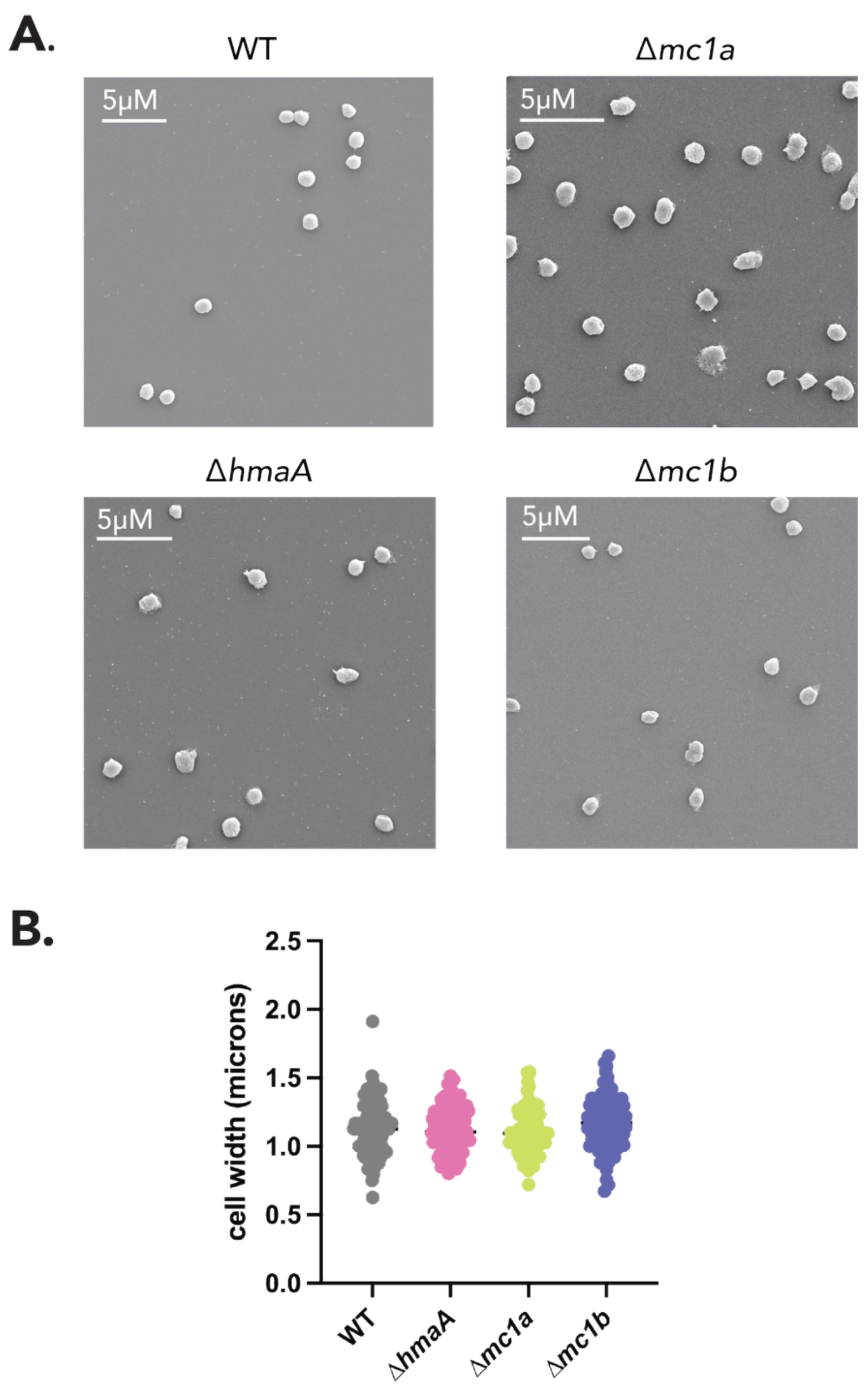

<u>Figure S4:</u> The morphology of mutants lacking any single DNA-binding protein (DBP) is indistinguishable from the parent strain. A. Scanning Electron Micrographs of each single DBP mutant strain as well as the parent strain (WWM60; denoted as wildtype or WT). Fields shown are representative of at least 10 randomly selected fields for each strain. **B.** Since *M.* acetivorans cells are spherical, cell width was measured for 100 cells of each strain by measuring the horizontal diameter of each cell (see Methods). The horizontal bar represents the mean of 100 replicates. No significant differences were observed between WT and any of the DBP deletion strains using a one-way ANOVA with Dunnett's multiple comparisons test.

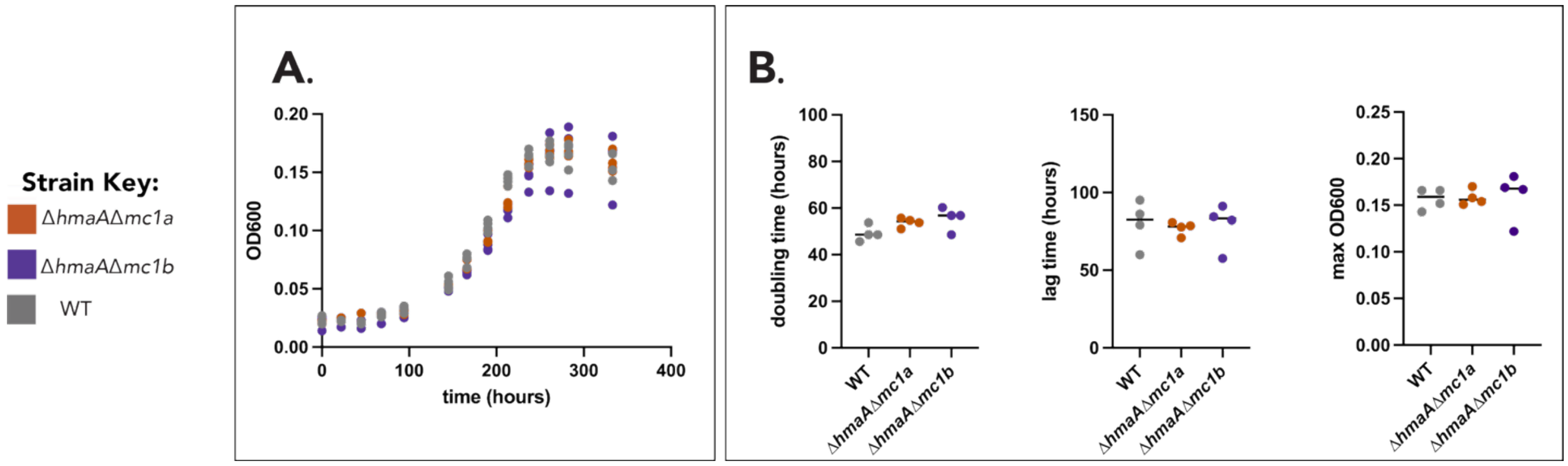

<u>Figure S5:</u> Double mutants are viable on acetate and have no growth defects. A. Growth curves of DNA-binding protein (DBP) double mutants (colored circles) and the parent strain (WWM60; denoted as wildtype or WT; gray circles) measured as a change in optical density (at 600 nm) over time in high-salt (HS) minimal medium supplemented with 40 mM sodium acetate as the growth substrate. Four replicates per strain are shown. **B.** Quantification of doubling time (in hours), lag time (in hours), cell yield (measured as maximum OD600) for replicates of each strain shown in **A**. The horizontal line represents the mean of the replicates. No significant differences in doubling time, cell yield, and lag time were observed using a one-way ANOVA with Dunnett's multiple comparisons test.

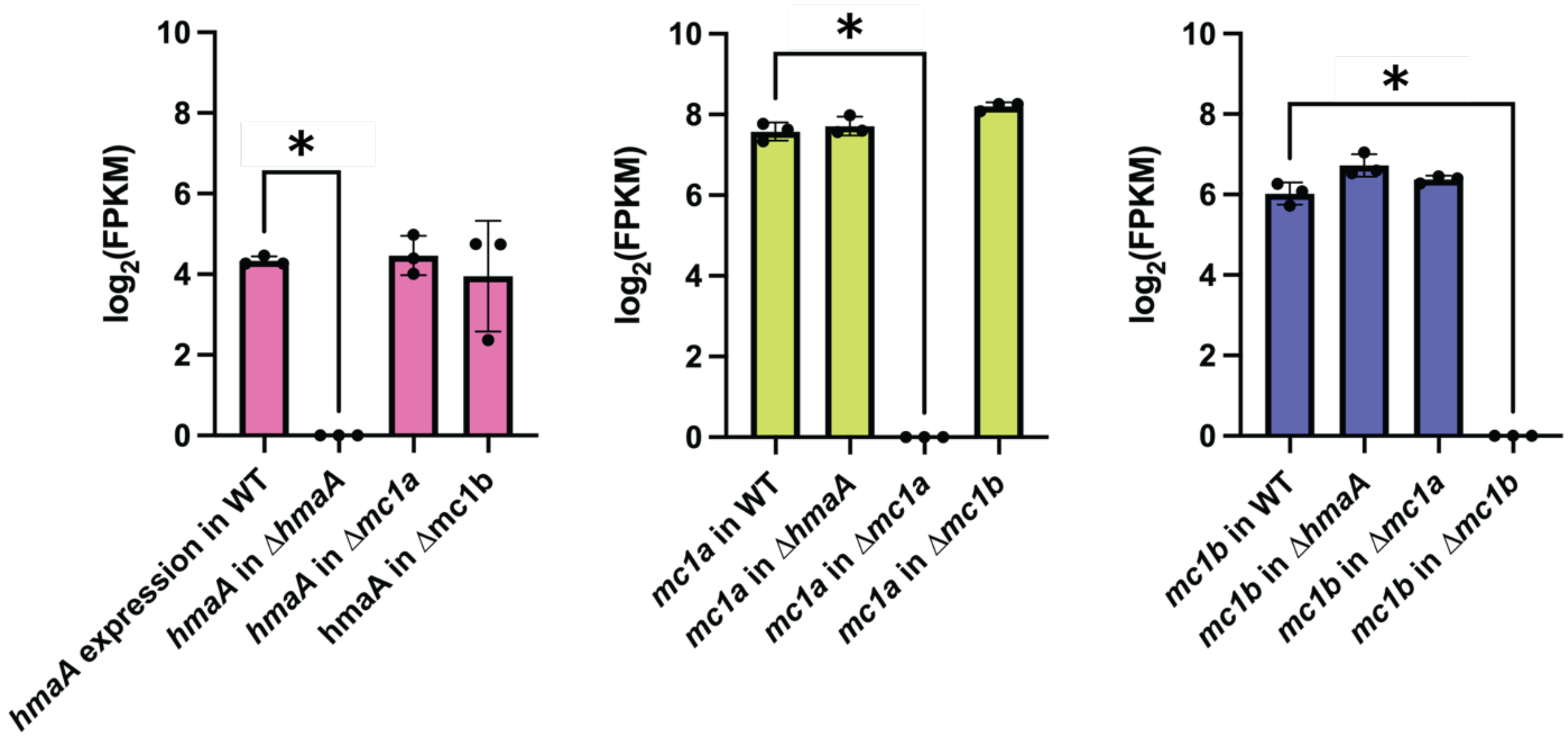

<u>Figure S6:</u> No evidence of transcriptional compensation by other DNA-binding proteins (DBPs) in individual DBP deletion backgrounds. Expression of the DBP encoding genes (colored bars; pink = *hmaA,* green = *mc1a,* blue = *mc1b*) in the parent strain (WWM60; denoted as wildtype or WT) and all the DBP single-knockout mutants. Bar height represents the mean of three biological replicates, individual points show values of each replicate and error bars represent one standard deviation. Expression of an individual DBP in the WT background was compared to expression of that same DBP in the single deletion strain backgrounds. Statistical significance calculated by one-way ANOVA with Tukey's multiple comparisons test; * indicates p-value ≤ 0.05.

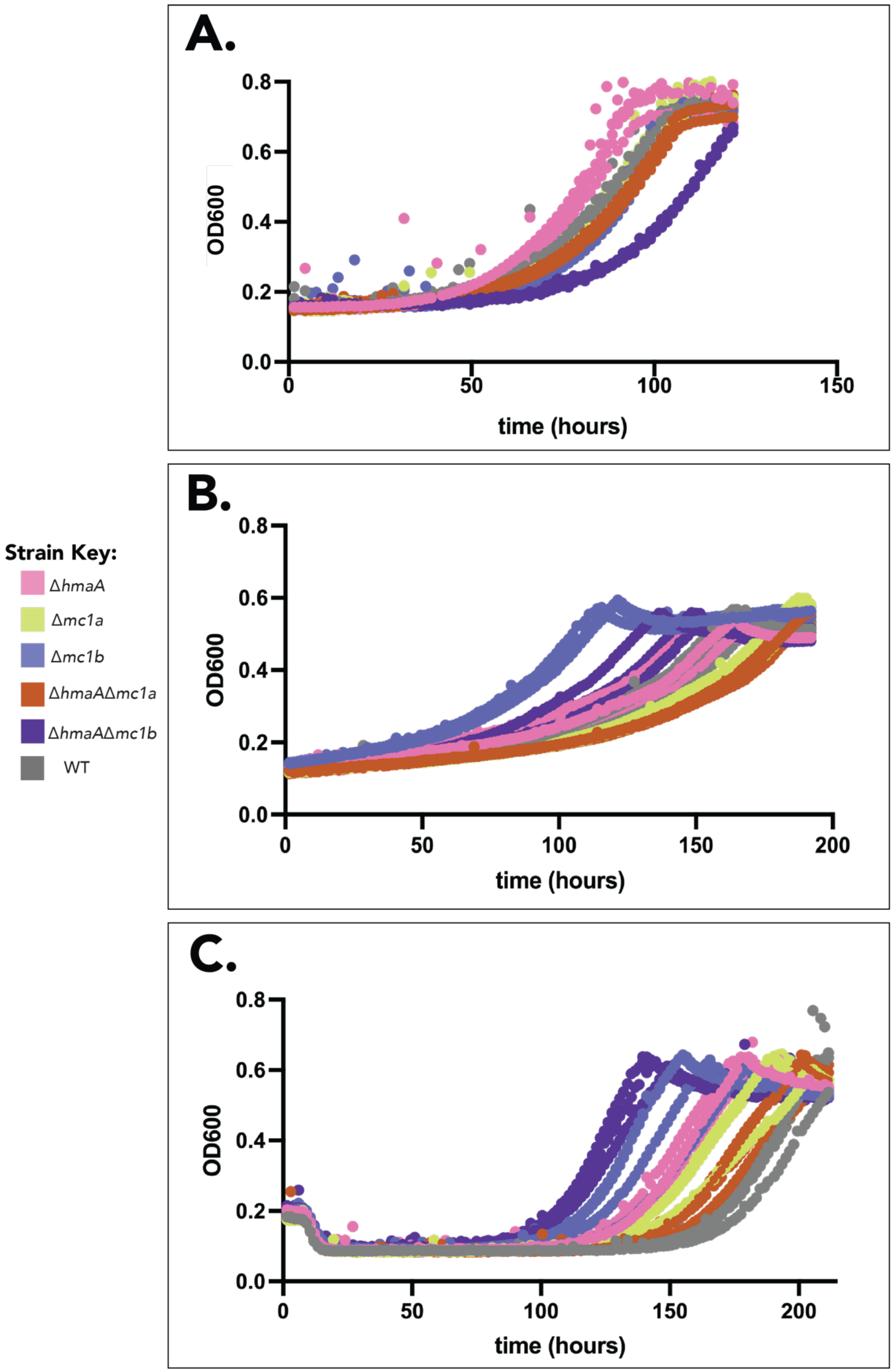
A.
B.
C.
OD600
time (hours)
Strain Key:
ΔhmaA
Δmc1a
Δmc1b
ΔhmaAΔmc1a
ΔhmaAΔmc1b
WT

<u>Figure S7:</u> Growth curves for at least three replicates of the DNA-binding protein (DBP) deletion mutants shown in Figure 3. Growth curves of DBP deletion mutants (colored circles) in comparison to the parental strain (WWM60, denoted as wildtype or WT in gray circles) measured as a change in optical density (at 600 nm) over time in high-salt (HS) minimal medium supplemented with 50mM trimethylamine as the growth substrate. Growth in HS media supplemented with 1 μM Mitomycin C (MMC) is shown in **A**.; growth at 40°C is shown in **B**.; growth after UV shock (see Methods) is shown in **C**. Growth parameters calculated from these curves are recorded in Table S5.

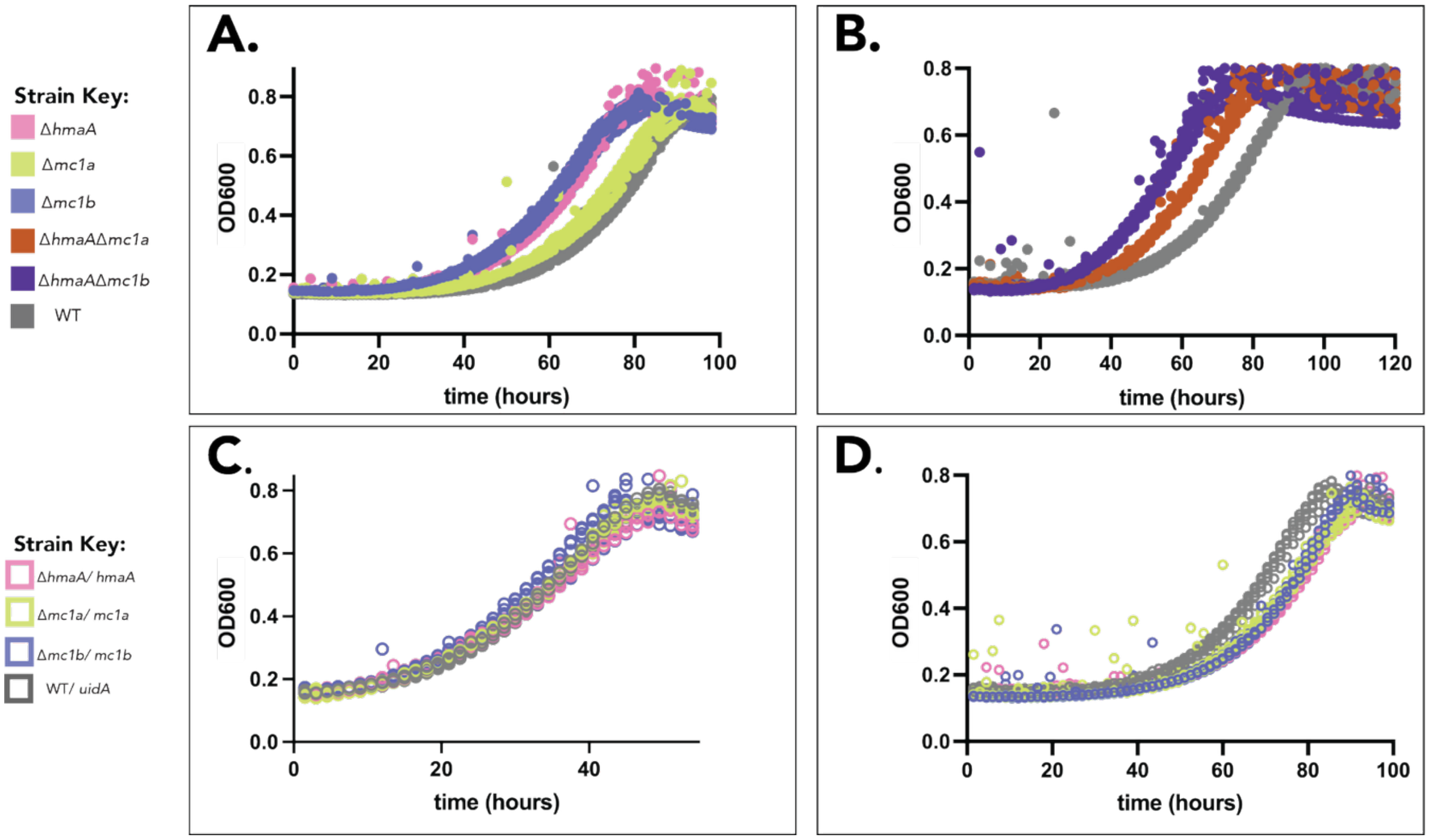

<u>Figure S8:</u> Growth curves for at least three replicates of the stationary exit experiments with the DNA-binding protein (DBP) mutant strains shown in Figure 4. A. Growth curves of the single DBP deletion mutants (colored closed circles) in comparison to the parental strain (WWM60, denoted as wildtype or WT in gray closed circles). **B.** Growth of the double DBP deletion mutants (colored closed circles) compared to the parental strain (WT, gray closed circles). All growth curves were conducted in high-salt (HS) minimal medium with 50 mM trimethylamine following 21 days incubation in stationary phase (see Methods). **C.** Growth of strains overexpressing DBPs in trans on a plasmid for each respective DBP-deletion background (colored open circles) compared to the parental strain expressing an equivalent amount of UidA in trans on a plasmid (gray open circles). Growth in panel **C** is after incubation in stationary phase for 1 day. **D.** Growth of the same strains as in panel **C** after stationary phase incubation for 21 days (See Methods). Growth parameters calculated from these curves are recorded in Table S6.

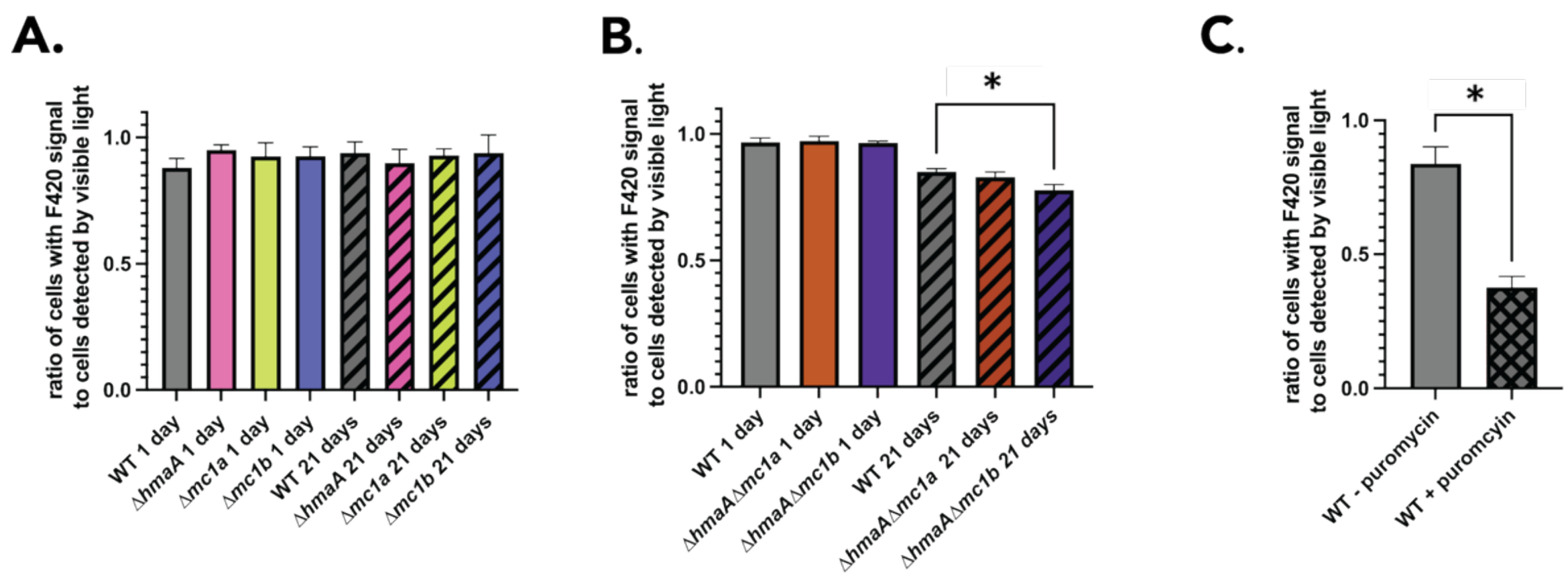

<u>Figure S9:</u> DNA-binding protein (DBP) deletion mutants and parent strain have similar numbers of intact cells after 21 days in stationary phase. A. Fraction of intact cells observed in at least 4 random fields of cells (total number of cells >300) for the parent strain (WWM60, denoted WT, gray bar) and the single DBP deletion strains (colored bars). The fraction of intact cells was determined by counting the number of cells with autofluorescence from factor 420 (F_{420}) that is present in the cytosol of intact cells, compared to the number of cells observed in the same field by visible light. Bars with solid color indicate observations after 1 day incubation in stationary phase. Bars with black hatched lines indicate observations after 21 days incubation in stationary phase. Statistical significance calculated by one-way ANOVA with Dunnett's multiple comparisons test; * indicates p-value ≤ 0.05. **B.** Fraction of intact cells observed in the double DBP mutant strains, following the same parameters described for panel **A**. **C.** Fraction of intact cells for the WT strain, which, upon reaching mid-exponential phase ($OD_{600} = 0.4$), were incubated for 72 hours with or without puromycin at a concentration of 2 µg/mL. Statistical significance calculated by Student's t-test; * indicates p-value ≤ 0.05.

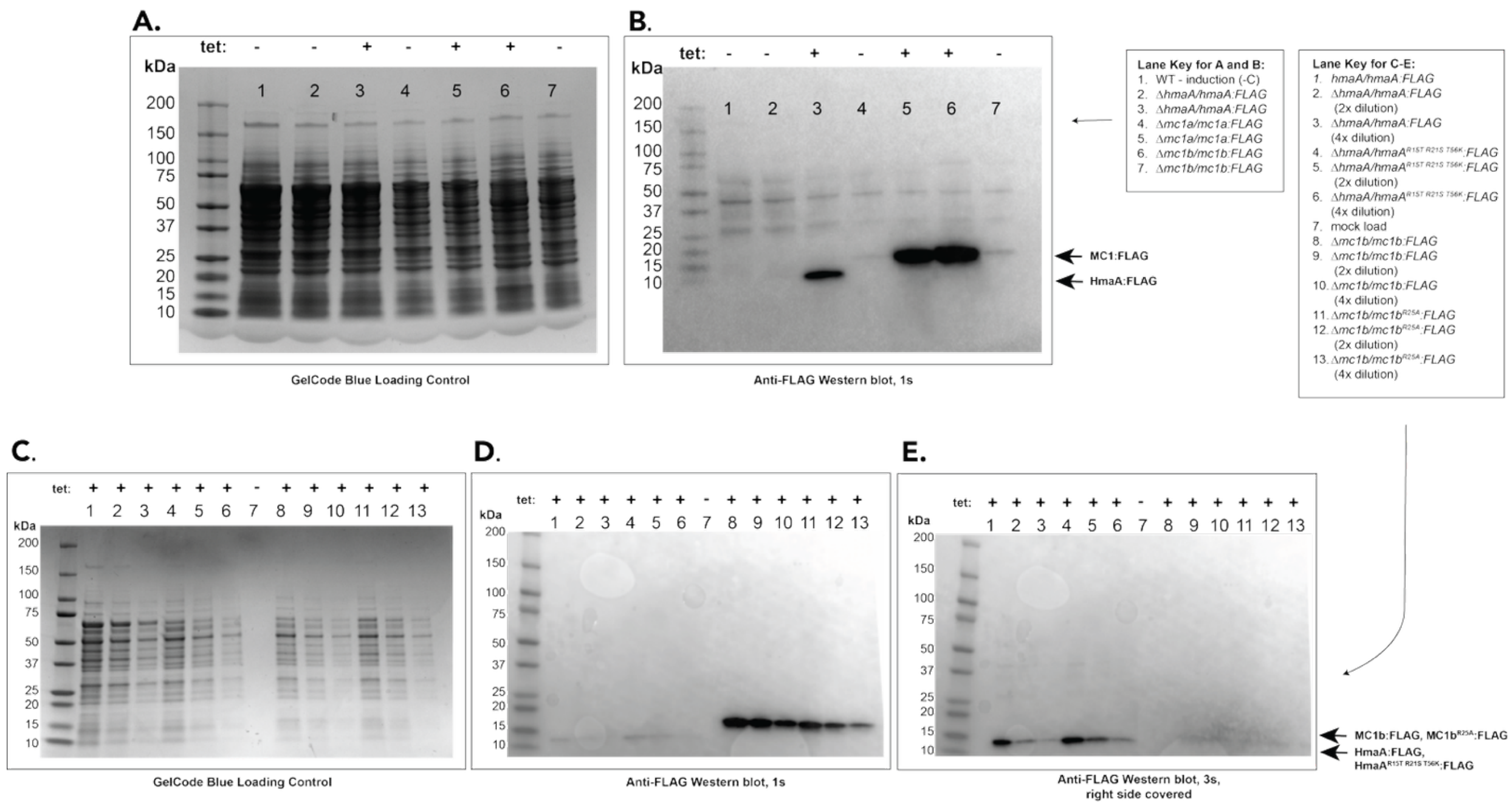

Figure S10: Complementation of DNA-binding protein (DBP) deletion mutants in trans with affinity-tagged DBP encoding genes. A. SDS-PAGE, **B.** Anti-FLAG Western blot with whole cell lysates for the DNA-binding protein (DBP) overexpression strains used in this study (see Fig. 4 and 5). A lane key indicating genotypes of strains in the gels shown in **A** and **B** is shown at right. Expected band size for HmaA:FLAG and MC1b:FLAG is 9kDa and 12kDa, respectively. Black labeled arrows indicate the expected locations of bands for these proteins. **C.** SDS-PAGE ,and **D.** Anti-FLAG Western blot with whole cell lysates for the point-mutated versions of the DBP overexpression strains used in this study (see Fig. 5). **E.** Shows the same blot as shown in panel **D**, with a longer exposure time and with a paper towel covering the right half of the blot to allow for brighter visualization of the HmaA bands. A lane key indicating genotypes of strains in each lane for gels in **C**, **D**, and **E** is shown in the top right corner of this figure. Expected band size for HmaA$^{R15T\ R21S\ T56K}$:FLAG and MC1b^{R25A}:FLAG is 9kDa and 12kDa, respectively. Black labeled arrows indicate the expected locations of bands for these proteins. For all panels, a key above the lanes indicates whether 100 µg/mL tetracycline (abbreviated "tet") was added to the culture medium to induce expression of the protein product (see Methods) and + indicates that tetracycline was added, - indicates that tetracycline was not added. Labels below each panel indicate the identity of the gel or blot depicted. For Western blots, exposure time in seconds (s) is given.

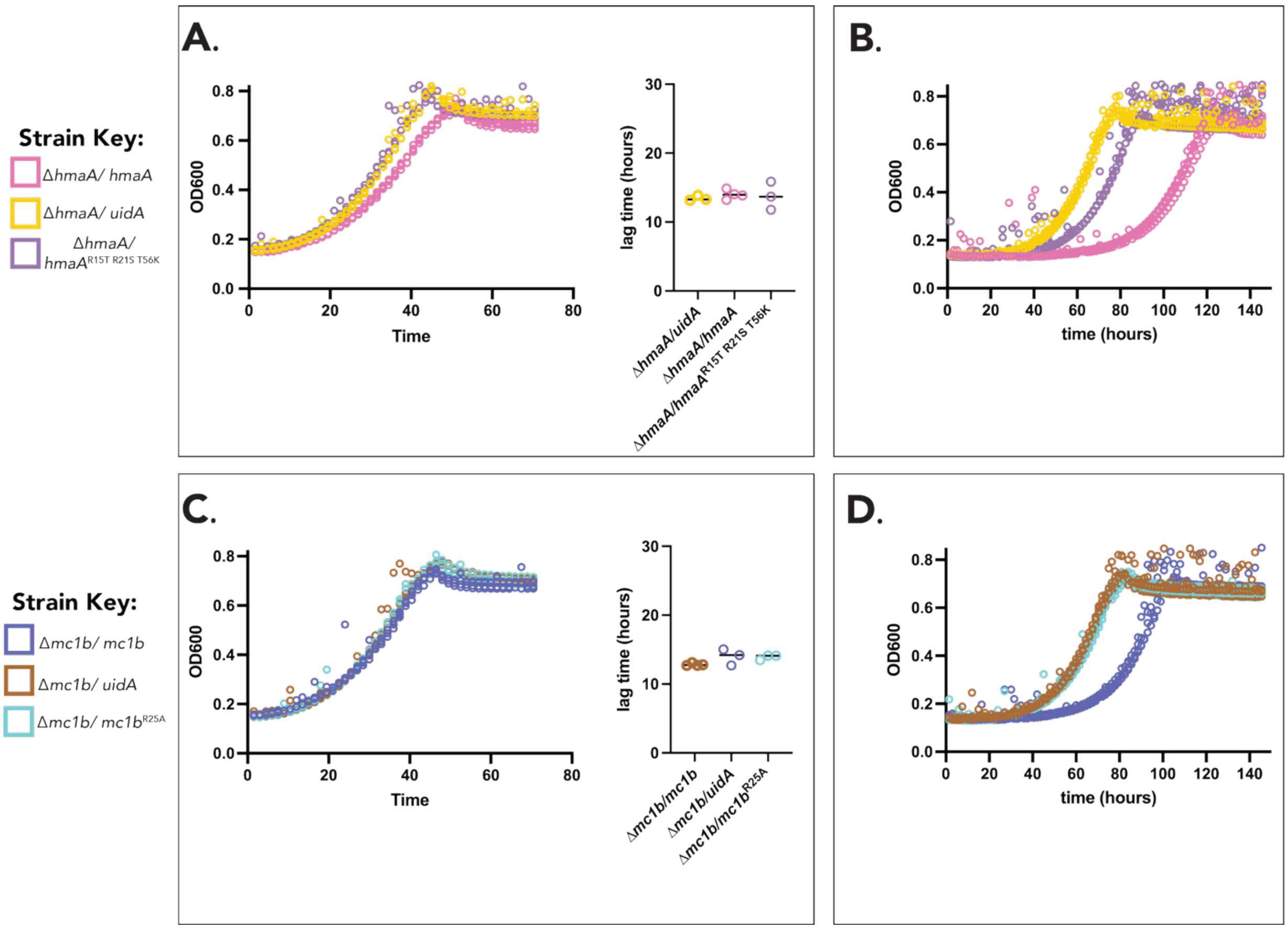

<u>Figure S11:</u> Growth curves for the stationary exit experiments with the DNA-binding protein (DBP) mutant strains shown in Figure 5. A. Growth of strains with plasmids encoding HmaA:FLAG (pink open circles), HmaA $^{R15T\ R21S\ T56K}$:FLAG (lavender open circles), or UidA (yellow open circles) in the Δ*hmaA* background after incubation in stationary phase for 1 day. All replicate growth curves are shown. Lag time is quantified for all replicates on the right. The horizontal bar represents the mean of at least three replicates. Any statistically significant ($p \leq 0.05$, one-way ANOVA, Dunnett's multiple comparisons test) differences are indicated with an asterisk. **B.** Growth of the same strains as panel **A.** after extended (28 days) stationary phase incubation. **C.** Growth of strains bearing over-expression plasmids for either WT MC1b:FLAG (blue open circles), MC1b ^{R25A}:FLAG (cyan open circles), or UidA (brown open circles) after incubation in stationary phase. **D.** Growth of the same strains as in panel **C.** after extended (28 days) stationary phase incubation. In all panels, at least three replicates for each strain are shown. Growth parameters calculated from these curves are recorded in Table S7.

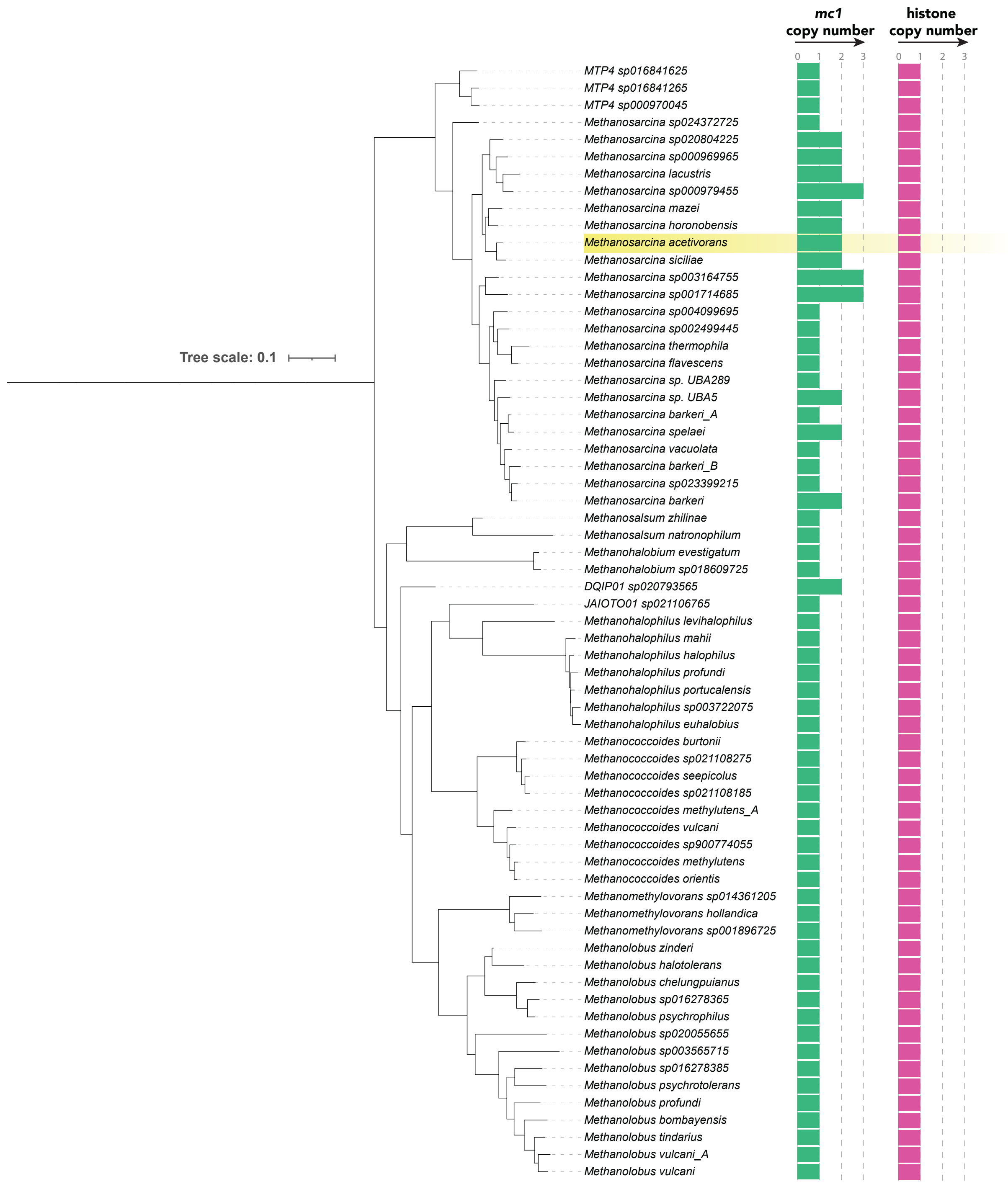

<u>Figure S12:</u> At least one copy of *mc1* and histone are maintained in the Family *Methanosarcinaceae*. Phylogeny of the *Methanosarcinaceae* from GDTB r214.0 aligned with a matrix showing the number of chromosomal copies of genes encoding the DBPs MC1 or histone as determined by a profile HMM search. The length of each bar indicates the number of copies of each DBP gene found in a given genome (see Methods). *Methanosarcina acetivorans,* the

organism used in this study, is highlighted in yellow. All genomes depicted exceed a genome completeness threshold of 97% by CheckM.
